# Size selection by a gape‐limited predator of a marine snail: Insights into magic traits for speciation

**DOI:** 10.1002/ece3.2659

**Published:** 2016-12-20

**Authors:** Elizabeth G. Boulding, María José Rivas, Nerea González‐Lavín, Emilio Rolán‐Alvarez, Juan Galindo

**Affiliations:** ^1^ Integrative BiologyUniversity of GuelphGuelphONCanada; ^2^ Departamento de Bioquímica, Genética e InmunologíaFacultad de BiologíaUniversidade de VigoVigoSpain; ^3^ ECIMAT, Estación de Ciencias Mariñas de TorallaUniversidade de VigoVigoSpain

**Keywords:** adaptation, fitness, life‐history evolution, *Littorina saxatilis*, morphological evolution, *Pachygrapsus marmoratus*, polymorphism, predator‐free space, selection—experimental, size‐limited predation

## Abstract

The intertidal snail *Littorina saxatilis* has repeatedly evolved two parallel ecotypes assumed to be wave adapted and predatory shore crab adapted, but the magnitude and targets of predator‐driven selection are unknown. In Spain, a small, wave ecotype with a large aperture from the lower shore and a large, thick‐shelled crab ecotype from the upper shore meet in the mid‐shore and show partial size‐assortative mating. We performed complementary field tethering and laboratory predation experiments; the first set compared the survival of two different size‐classes of the crab ecotype while the second compared the same size‐class of the two ecotypes. In the first set, the large size‐class of the crab ecotype survived significantly better than the small size‐class both on the upper shore and in the laboratory. In the second set, the small size‐class of the crab ecotype survived substantially better than that of the wave ecotype both on the upper shore and in the laboratory. Shell‐breaking predation on tethered snails was almost absent within the lower shore. In the laboratory shore crabs (*Pachygrapsus marmoratus*) with larger claw heights selected most strongly against the small size‐class of the crab ecotype, whereas those with medium claw heights selected most strongly against the thin‐shelled wave ecotype. Sexual maturity occurred at a much larger size in the crab ecotype than in the wave ecotype. Our results showed that selection on the upper shore for rapid attainment of a size refuge from this gape‐limited predator favors large size, thick shells, and late maturity. Model parameterization showed that size‐selective predation restricted to the upper shore resulted in the evolution of the crab ecotype despite gene flow from the wave ecotype snails living on the lower shore. These results on gape‐limited predation and previous ones showing size‐assortative mating between ecotypes suggest that size may represent a magic trait for the thick‐shelled ecotype.

## 1 Introduction

Spatial variation in the strength of selection by different species of predators has been shown to have a key role in the evolution of ecotypes and species (Calsbeek & Cox, 2010; Endler, 1977, 1986; Langerhans, Layman, Shokrollahi, & DeWitt, 2004; Mezquida & Benkman, 2014; Schluter, 2000). Divergent selection pressures on phenotypic traits caused by spatial variation in the abundances of predators are believed to be sufficiently strong to counteract the homogenizing effect of gene flow (Faria et al., 2014; Kisel & Barraclough, 2010; Tigano & Friesen, 2016) and promote genetic differentiation of populations (Jones et al., 2012; Seehausen et al., 2014). Higher predation on immigrants from a spatially separated and contrasting habitat, referred to as immigrant inviability, can represent a particularly important barrier to gene flow between two incipient ecotypes or species (Ingley & Johnson, 2016; Nosil, 2012; Nosil & Crespi, 2004; Nosil, Vines, & Funk, 2005; Schluter, 2000). Such a barrier could more easily lead to reproductive isolation if an antipredator trait under strong spatially divergent selection (e.g., body size) is also a major cue contributing to nonrandom mating. If the trait under divergent selection and the mating cue trait are pleiotropic expressions of the same gene(s), then recombination cannot break up their association (Servedio, Van Doorn, Kopp, Frame, & Nosil, 2011). Such traits have been classified to as a “magic” traits (Gavrilets, 2004). Body size has often been suggested to be a magic trait (e.g., sticklebacks, seahorses, amphipods, and intertidal snails; Servedio et al., 2011). However, even classifying body size as a magic trait has been controversial because there is little evidence for any gene controlling both it and mating cues (Smadja & Butlin, 2011). Also uncommon are quantitative estimates of divergent selection and of assortative mating with respect to body size from the same population (Haller, De Léon, Rolshausen, Gotanda, & Hendry, 2012; Servedio, Van Doorn, Kopp, Frame, & Nosil, 2012).

As a starting point, it is important to estimate the strength of selection on traits under divergent predator‐driven selection. Considerable progress has been made in measuring selection by predators in field (Reznick & Ghalambor, 2005; Reznick, Shaw, Rodd, & Shaw, 1997), laboratory (Vamosi, 2002), and mesocosm experiments (Rennison, Heilbron, Barrett, & Schluter, 2015). For example, divergent selection by different guilds of predators (Reimchen, 1994) is sufficient to cause genetic differentiation at a major locus affecting defensive armor among different ecotypes of threespine sticklebacks (Marchinko, 2009). On the other hand, most predator‐resistant traits such as body size are known to be quantitative traits determined by multiple minor loci and by the environment (Falconer & Mackay, 1996). Size is expected to be particularly important for armored prey that can reach a size refuge from their major predator (Paine, 1976; Vermeij, 1987). A common strategy for armored prey with major predators that are gape‐limited is to grow too large to fit into their predator's feeding appendage (Miehls, Peacor, & McAdam, 2014; Vermeij, 1987).

Analytical models of the evolution of nonmagic quantitative traits under spatially divergent selection pressures often assume that the environmental gradient is linear (Kirkpatrick & Barton, 1997; Miller & Zeng, 2014). Such models are not designed for step‐like gradients often seen under predator‐driven selection when a predator only occurs in part of its prey's habitat (Boulding et al., 2007). Theoretically, a quantitative trait such as body size will show a step‐like shift to a new optimum in response to predator‐driven selection only if directional/stabilizing selection toward the optimum is high, and the size of the habitat containing the predator is sufficiently large relative to the prey's lifetime dispersal distance (Slatkin, 1978).

An example of incipient ecological speciation in response to predator‐mediated selection is believed to occur between ecotypes of the marine snail *Littorina saxatilis* on exposed shores of northwestern Spain (reviewed in Rolán‐Alvarez, 2007). The upper intertidal is inhabited by a large crab‐resistant ecotype (formerly RB) that possesses a thick, color‐banded shell with ridges whereas the lower intertidal is inhabited by a small wave‐resistant ecotype (formerly SU) that possesses a smooth, unbanded shell with a large aperture (Johannesson, Johannesson, & Rolán‐Alvarez, 1993). These shell characters are highly heritable and phenotypic plasticity represents a minor component of the phenotypic variation (Carballo, Garcia, & Rolán‐Alvarez, 2001; Conde‐Padin, Caballero, & Rolán‐Alvarez, 2009; Conde‐Padin, Grahame, & Rolán‐Alvarez, 2007; Hollander & Butlin, 2010; Galindo, Martínez‐Fernández, Rodríguez‐Ramilo, & Rolán‐Alvarez, 2013). The crab ecotype is believed to be adapted to predation by the marbled shore crab *Pachygrapsus marmoratus* which hides in crevices on the upper shore (Silva, Brazoa, Hawkins, Thompson, & Boaventura, 2009). In a preliminary laboratory experiment, this crab consumed more wave ecotype than crab ecotype snails, and did not consume any of the largest crab ecotype snails (Rolán‐Alvarez, Johannesson, & Erlandsson, 1997). The wave ecotype is believed to be adapted to the strong wave action on the lower shore. A preliminary laboratory experiment showed lower dislodgement rates of wave ecotype snails than those of crab ecotype snails when attached to a glass surface accelerated underneath sea water (Rolán‐Alvarez et al., 1997). Reciprocal transplants of marked snails of the crab and wave ecotypes suggest the action of divergent selection because ecotypes had higher survival in their native habitat than in the contrasting habitat (Cruz, Vilas, Mosquera, & García, 2004a; Cruz, Vilas, Mosquera, & García, 2004b; Rolán‐Alvarez et al., 1997). Unfortunately transplant experiments do not permit the direct estimation of the magnitude of natural selection because transplanted snails tend to return to their home tidal level, so missing snails cannot be assumed to be dead (Cruz et al., 2004b). Field tethering experiments have the advantage that the shell fragments of dead snails remain attached to the tethering line, often allowing identification of the predator (Behrens Yamada & Boulding, 1996; Boulding, Holst, & Pilon, 1999) and have not been previously conducted on these ecotypes.

Studies using molecular markers to compare the two ecotypes at multiple sites have consistently shown that population samples of the two ecotypes from a particular shore are more similar to each other than to a sample of the same ecotype from a distant site (Butlin et al., 2014; Galindo, Morán, & Rolán‐Alvarez, 2009; Galindo et al., 2013; Quesada et al., 2007; Rolán‐Alvarez et al., 2004; Westram, Panova, Galindo, & Butlin, 2016; Westram et al., 2014). Partial reproductive isolation has been demonstrated. The two ecotypes meet and mate in the middle intertidal, but field and laboratory observations show partial size‐assortative mating, and a low frequency of interecotype matings (Conde‐Padin, Cruz, Hollander, & Rolán‐Alvarez, 2008; Erlandsson, Kostylev, & Rolán‐Alvarez, 1999; Erlandsson & Rolán‐Alvarez, 1998; Johannesson, Rolán‐Alvarez, & Ekendahl, 1995; Rolán‐Alvarez, Erlandsson, Johannesson, & Cruz, 1999) but no postzygotic isolation (Saura, Martinez‐Fernandez, Rivas, Caballero & Rolán‐Alvarez, 2011). Therefore, shell size represents a potential magic trait in these ecotypes (Servedio et al., 2011); divergent selection on size between the upper and lower shores increases the genetic divergence in size and consequently the level of reproductive isolation between ecotypes.

In this study, we test predictions from the hypothesis that strong selection for attainment of a size refuge from predation by the marbled shore crab is sufficient to explain the genetic divergence that has been previously documented between the upper shore crab ecotype and the lower shore wave ecotype of *L. saxatilis* from northwestern Spain (Rolán‐Alvarez, Austin, & Boulding, 2015). We first quantified the phenotypic divergence in shell shape, shell thickness, and life‐history traits between the ecotypes across their natural size range. We then present data from two field tethering experiments that compare the rate of mortality and strength of natural selection for two discrete categories of prey tethered at different tidal heights. We also performed two complementary laboratory experiments offering the same two prey categories to a broad size range of the marbled shore crab. In addition, we used our field estimates of the direction and magnitude of natural selection to estimate the minimum length (perpendicular to the water’s edge) over which this poorly dispersing, live‐bearing snail could adapt to this predatory crab. Finally, we evaluate the evidence for shell size being a classic magic trait in this system.

## 2 Methods

### 2.1 Experimental design overview

To separate the effect of prey size from that of prey shell thickness, we first measured crab predation by the marbled shore crab *P. marmoratus* on *Littorina saxatilis* using two prey categories differing mostly in shell size (crab ecotype of 4 vs. 9 mm) and then used two prey categories differing mostly in shell thickness (crab ecotype of 4 mm vs. wave ecotype of 4 mm). These two sets of prey categories were each used in one field experiment and one parallel laboratory experiment. In the field experiment, we tethered snails to the rocks along three transects at discrete tidal levels, so that shell‐crushing predation rates for each prey category could be estimated from shell fragments that remained attached to the tether. In the laboratory, the crabs were kept in individual aquariums and offered snails of each prey category, so that relative predation rates could be determined as a function of crab claw height (CH).

### 2.2 Sampling

**Figure 1:**
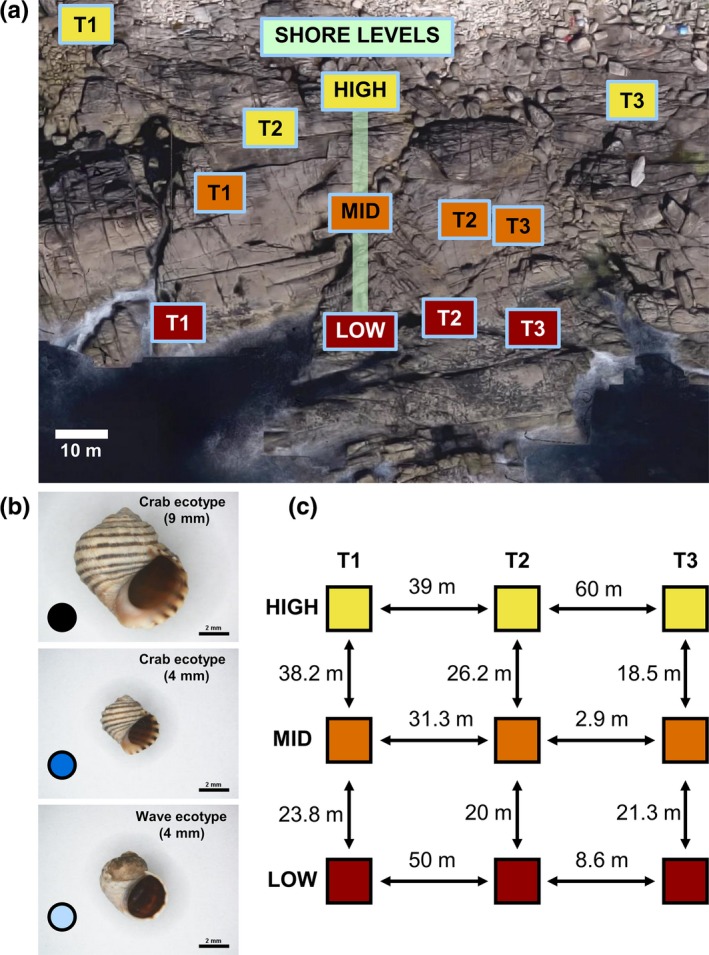
Experimental design of field tethering experiments. (a) Location of the three transects (T1, T2, and T3) and shore levels (High, Mid, and Low) at Silleiro (Oia, NW Spain). (b) Three prey categories of *Littorina saxatilis* of which only two were used per experiment: large crab ecotype (mean shell length 9 mm), small crab ecotype (4 mm), and wave ecotype (4 mm). (c) Distance in meters among the three different tidal levels of each of the three transects

Samples of *L. saxatilis* were taken multiple times from two consistent locations (Figure 1, Appendix S1) between October 2013 and June 2014 at Cabo Silleiro (42°06′17″N; 8°53′56″W; Galicia, Spain), a well‐studied site for these ecotypes (Galindo et al., 2009, 2013; Rolán‐Alvarez, 2007). The samples were then sorted using digital calipers into the following three prey categories: (1) 4‐mm wave –ecotype (3–5 mm, shell apex to columella length (SL)), (2) 4‐mm crab ecotype (3–5 mm SL), and (3) 9‐mm crab ecotype (8–10 mm SL). There was no prey category for the 9‐mm size class of the wave ecotype because such individuals are extremely rare. These snails were used for the laboratory crab predation and field tethering experiments. Samples from the same two locations but encompassing a broader size range were used to quantify the phenotypic divergence between the two ecotypes.

Twenty male marbled shore crabs (*P. marmoratus*) were collected near ECIMAT marine station (University of Vigo, Spain) and held in individual aquaria (Fig. S1a‐c). Digital calipers were used to measure the carapace width (CW), CH, claw or fixed finger length (CL), and dactyl or movable finger length (DL) of each crab used in the predation experiments so that claw gape size could be estimated (Behrens Yamada & Boulding, 1998). The crabs were classified into five size‐classes with respect to CH (CW): “small” 7 mm (15–19 mm), “medium” 9 mm (20–24 mm), “large” 11 mm (25–29 mm), “very large” 13 mm (30–34 mm), and “extra large” 16 mm (35–40 mm). The same 20 crabs were used for both laboratory predation experiments. Sampling details and maintenance of the snails and crabs in an ECIMAT laboratory is described in Appendix S1.

### 2.3 Phenotypic divergence between ecotypes

To evaluate the potential traits under natural selection by the predatory marbled shore crab, we quantified the differences in shell, body, and life‐history traits between the crab and wave ecotypes across their size ranges. From the snails maintained in the laboratory (<15 days), 10 random snails within each 0.5‐mm interval from 3 to 13 mm in shell length for the crab ecotype and from 3 to 6 mm for the wave ecotype were selected. Then, shell size and shape (Fig. S1d‐e; geometric morphometric analysis from photographs; Carvajal‐Rodriguez, Conde‐Padin, & Rolán‐Alvarez, 2005; Rohlf, 2015), dry shell weight, shell thickness, dry body weight, gender, and maturity were recorded for each snail. This methodology is described in detail in Appendix S2. First, each recorded trait was tested with an unpaired *t* test that assumed unequal variances to see if it differed significantly between ecotypes. Second, all significant traits were then examined to see whether they showed microgeographic divergence between the two ecotypes as defined by Richardson, Urban, Bolnick, and Skelly (2014).

### 2.4 Laboratory predation experiments

#### 2.4.1 4‐mm and 9‐mm crab ecotype predation experiment

In the first laboratory predation experiment, 10 snails of each of these size classes of the crab ecotype were introduced onto the bottom of each aquarium (*N* = 20) containing an individual crab (Fig. S1c). Approximately every 2 days, all live snails and shell fragments were recovered from each aquarium and each classified as AU (Alive Undamaged), AP (Alive Peeled), AC (Alive Chipped), DU (Dead Undamaged), DC (Dead Chipped), DP (Dead Peeled), or M (Missing because of counting errors, see Appendix S1). The snails were replaced with 10 live snails with undamaged shells of each of the two prey classes. A total of seven trials at 2‐ to 3‐day intervals were conducted (9–25 April 2014). The mean percent mortality rate per crab per trial for each prey category was then calculated by averaging all seven trials for all crabs in each of the five crab size‐classes.

#### 2.4.2 4‐mm crab ecotype and wave ecotype predation experiment

In the second laboratory predation experiment, 10 snails of each ecotype of the size class 4 mm were introduced into each aquarium (*N* = 20) containing an individual crab following the same laboratory and statistical methodology used in the previous experiment. The experiment began on 30 April and ended on 11 May 2014 after four trials had been conducted at intervals of 2–4 days.

### 2.5 Field tethering experiments

The experimental design of the tethering experiment carried out at Cabo Silleiro consisted of three randomly placed vertical transects, each with three tidal levels or microhabitats (High, Mid, and Low) (Figure 1a,c). The Low level was situated at the upper limit of the blue mussel belt (*Mytilus galloprovincialis*), Mid was placed below the upper limit of the acorn barnacle zone (*Chthamalus stellatus*) and High was in the splash zone just above the barnacles where the marble shore crab density is greatest (E. G. Boulding, personal observations). At each tidal level of each transect, we tethered 20 snails to screws attached to the rock following Behrens Yamada and Boulding (1996); see Appendix S3).

#### 2.5.1 4‐mm and 9‐mm crab ecotype tethering experiment

In this field experiment, we used the same prey categories (4‐mm and 9‐mm crab ecotype) that were used in the first laboratory experiment. Twenty snails were tethered at the Mid and High tidal levels of each of the three transects. To avoid confounding microhabitat (<1 m) effects with snail prey type, an individual of the 4‐mm size class and an individual of the 9‐mm size class were tethered alternately to 20 successive screws (Fig. S3). A total of six replicate trials were carried out between 12 and 29 November 2013. At the end of each replicate trial, all tethered snails were replaced with new freshly tethered snails. Live snails, shell fragments, and pieces of epoxy still attached to the previous replicate's tethering lines were placed individually into containers and kept for later comparison with shell fragments from the laboratory experiments (Fig. S3c). Recovered snails and their tethering lines were classified into 12 categories (see Appendix S1 for a more complete description), and for the main statistical analysis, we used three categories: “1” for not killed, “0” for likely to have been killed by crabs, and the rest were coded as missing data.

#### 2.5.2 4‐mm crab ecotype and wave ecotype tethering experiment

In this field experiment, we used the same two prey categories (4‐mm crab ecotype and 4‐mm wave ecotype) as in the second laboratory experiment. The experiment was conducted in the spring when the average wave height was lower (20 April to 12 June 2014); therefore, it was possible to tether the snails at the three tidal levels (High, Mid, and Low) of each of the three transects. One problem recognized only after including the Low level was that it could only be reached during monthly periods of large tidal amplitude. Consequently, in this second experiment the snails were replaced only when they were damaged or dead every 3–15 days for a total of six replicate trials. The rest of the methodology was the same as in the previous experiment.

### 2.6 Statistical analysis

Statistical analyses were performed with SPSS (Released 2014. IBM SPSS Statistics for Windows, version 23.0.; IBM Corp., Armonk, NY, USA) or with Systat (Released 2009. Systat Software Statistics for Windows, version 13.0005, San Jose, CA, USA). Akaike's information criterion corrected for small sample sizes (AIC_c_) was used to decide whether one statistical model fit the data significantly better than another (Akaike, 1973). A more complex model was considered to better fit the data if the difference in its AIC_c_ was more than two units smaller than the alternative model (Anderson, 2008).

#### 2.6.1 Phenotypic traits of the two ecotypes

To estimate the relationships among the phenotypic variables, linear regression analysis was performed separately for each ecotype for all traits. Three different size ranges were used: (1) the entire size range sampled from each ecotype, (2) the 3–5 mm shell length, and (3) the 8–10 mm shell length (only for the crab ecotype). Differences in size at sexual maturity were tested using only the size range between 3 and 6 mm in shell length that had been collected for both ecotypes. A Fisher's exact test was used to test for an association between ecotype and the number of immature males and of mature males. A second Fisher's exact test of the association between ecotype and the number of juveniles and of mature females was performed.

#### 2.6.2 Phenotypic traits of the crabs

Carapace width and three claw measurements taken from the crabs used in the laboratory experiments were used in an all possible subsets multiple regression. This was done to confirm whether CH was the best predictor of the selection differential for a particular crab as suggested by Behrens Yamada and Boulding (1998). The analysis used Mallows’ Cp as implemented in the regression module in Systat to select the best combination of variables.

#### 2.6.3 Survival analysis of different types of snail prey categories

In the laboratory predation experiments, logistic regression was used to compare the number of individuals from each of the two prey categories that remained alive with the number that were dead because of shell‐breaking predation. This analysis was carried out with GENLIN (SPSS) with binary fate as the dependent variable, snail prey category as a categorical independent variable, and crab CH as a continuous independent variable.

In the field tethering experiment where all variables were categorical, we first used hierarchical log‐likelihood analysis in Systat to find the smallest set of interaction terms that fit the data from the set of variables (transect, tide level, prey category, and binary fate with trial used as a structuring variable). We then used the log‐likelihood ratio chi‐square analysis (*G* statistic) to test for biologically relevant associations among these terms.

#### 2.6.4 Univariate linear selection differentials and gradients

To quantify positive selection for a trait (either shell size or shell thickness), the univariate linear selection differential (*S*) and standardized selection differential (*i*) were calculated. This was performed separately for each crab in the laboratory predation experiments and for each combination of transect and tidal level in the tethering experiments. There were only two prey categories used in any experiment. The first set of tethering and field experiments with the 4‐mm and 9‐mm prey categories of the crab ecotype used the mean shell length of each category (4.02 and 8.97 mm, respectively, Table 1) as the phenotypic trait values whereas the second set of experiments with the 4‐mm prey categories of the crab and wave ecotypes used the mean shell thickness of each category (0.250 and 0.150 mm, respectively, Table 1). With only two possible trait values, S was calculated by subtracting the mean trait value at the beginning of each trial (with equal prey type numbers, this was the average of the two trait values) from the mean after the trial (obtained by multiplying the trait value for a prey category by the frequency of the number of surviving prey in that category) following Pakes and Boulding (2010). Subsequently, *i*, the standardized selection differential, could be calculated by dividing *S* by the trait phenotypic standard deviation (PSD) that had been calculated from the frequencies of the two prey categories at the beginning of each trial (Pakes & Boulding, 2010). Note that by assuming all the individual snails within our narrowly defined prey category had the mean trait value for that category, we did not have to measure each individual snail. This allowed us to use large numbers of prey individuals in our experiments which gave us high statistical power to detect positive selection. The main disadvantage of the prey category method of calculating selection differentials (Pakes & Boulding, 2010) is that it did not enable us to separate the univariate selection gradient into linear (directional) and quadratic (stabilizing) components (Fairbairn & Reeve, 2001).

**Table 1:** Microgeographic divergence in shell and life‐history traits among two ecotypes of *Littorina saxatilis* including that between the three prey categories used in two sets of laboratory predation and field tethering experiments

Ecotype (Size class)	Abbreviation	Shell length (mm) Mean ± *SE*	Shell weight (mg) Mean ± *SE*	Body weight (mg) Mean ± *SE*	SW/BW ratio^a^Mean ± *SE*	Shell thickness (mm)Mean ± *SE*	Aperture shape (RW1)Mean ± *SE* (×10^−2^)
Wave 3‐ to 5‐mm prey^b^	4‐mm wave	4.00 ± 0.571	17.4 ± 8.38	3.62 ± 1.88	4.93 ± 0.579	0.150 ± 0.0266	8.57 ± 0.824
Crab 3‐ to 5‐mm prey^b^	4‐mm crab	4.02 ± 0.566	18.8 ± 9.11	1.04 ± 0.407	18.45 ± 6.017	0.250 ± 0.0567	−2.46 ± 0.693
Crab 8‐ to 10‐mm prey^b^	9‐mm crab	8.97 ± 0.585	213 ± 49.9	11.5 ± 3.53	19.1 ± 2.89	0.465 ± 0.0762	−4.43 ± 1.05
Wave 3‐ to 6‐mm^c^	Population	4.49 ± 0.857	24.0 ± 12.6	5.14 ± 2.74	4.81 ± 0.0880	0.160 ± 0.0354	9.15 ± 0.632
Crab 3–13 mm^d^	Population	7.98 ± 2.86	199 ± 171	10.6 ± 9.59	19.7 ± 0.312	0.420 ± 0.125	−2.88 ± 0.391
Divergence^e^crab–wave	Wrights (units per meter)	0.333 to 0.698^f^	0.293 to 0.615^f^	0.173 to 0.364^f^	0.564 to 1.19^f^	0.465 to 0.977^f^	−0.455 to −0.956^f^

a SW = shell weight of dried empty shell divided by BW = dried body weight.

b One of three prey categories used in field tethering and laboratory experiments *n* = 40.

c Wave ecotype population sample used in quantification of trait divergence between ecotypes *n* = 60.

d Crab ecotype population sample used in quantification of trait divergence between ecotypes *n* = 200.

e Standardized spatial scale of divergence and adaptation between the “population” of each ecotype (in Wrights, defined as “number of trait standard deviations separating two samples per lifetime dispersal neighborhood” (meters).

f Divergence among population ecotypes significant (two‐sample *t* test, *p *<* *0.001).

### 2.7 Theoretical versus observed scales of local adaptation

Our objective was to estimate the minimum length, perpendicular to the water's edge, of the upper shore that must be inhabited by the predatory marbled shore crab to ensure the maintenance of the crab ecotype and the wave ecotype. This is mathematically equivalent to finding the minimum “characteristic length” of the zone (Slatkin, 1978). This model assumes that there is divergent phenotypic selection on the upper and the lower shore that will result in the evolution of a cline in predator‐resistant traits. Finally, we compared the theoretical characteristic length with that of the observed spatial scale of microgeographic divergence of the two ecotypes.

#### 2.7.1 Characteristic length equation used to estimate minimum width of zone required

Slatkin (1978) described a model of a step cline in a quantitative trait such as might occur when an environmental gradient causes an abrupt change in the optimum of a phenotypic trait as one ascends up the beach in the intertidal zone. His analytical solution showed that detectable adaptation to local conditions would occur when a change in the optimum occurred over distances greater than *L*_c_, the characteristic length. Substitute equation 11 of Slatkin (1978) into his equation 23 but using the notation of Boulding et al. (2007):

(1) $$L_{C}=\frac{d}{\sqrt{\left(h^{2}V_{z}\right)/\left(V_{z}+\omega^{2}\right))}}$$

where: *d* is *SD* of lifetime dispersal displacements, *h* ^2^ is the heritability of the trait, *V*_*z*_ is the phenotypic variance of the trait, and ω^2^ is inversely proportional to the strength of stabilizing selection. The model assumes that all parameters are constant throughout the cline, that stabilizing selection toward the local optimum (crabs present or not) is weak, and that the range of the optima along the environmental gradient is small and that dispersal was symmetrical (up and down the shore) (Slatkin, 1978).

#### 2.7.2 Estimation of the model parameters

Parameters for equation (1) were estimated for *L. saxatilis* using similar field and statistical methods to those described in Boulding et al. (2007). *d* was estimated using dispersal distances from the literature. The median migration distance for wave ecotypes transplanted back to the low shore at Silleiro was 2 m/month and that for the crab ecotype transplanted back to the high shore at a different site was 1.5 m/month (Erlandsson, Rolán‐Alvarez, & Johannesson, 1998). These median migration distances were converted to *d* by multiplying by 2.8 (Wright, 1969).

A value of *h* ^*2*^ *= *0.3 was used for both size and shell thickness. This value was the average for *Littorina* spp. from the literature (range 0.1–0.5; average 0.3; for thickness estimated as shell weight, see Boulding & Hay, 1993; for size or shape from the same population, see Carballo et al., 2001; Conde‐Padin et al., 2007; Galindo et al., 2013). *V*_z_ *= *1 because stabilizing selection, ω, was estimated in units of PSDs. Our field tethering experiments were used to estimate ω in trait PSD units using the Gaussian fitness equation (equation (2) in Boulding & Hay, 2001):

(2) $$W_{x}=e^{-({\left(z_{x}-\theta_{x}\right)}^{2}/\left(2\omega_{x}^{2}\right))}$$

where *W*_*x*_ is the relative fitness of a prey category *x* with trait value *z*_*x*_ at the high tidal level of a transect experiencing high shell‐breaking predation in one of the two field tethering experiments and θ_*x*_ is the optimal trait value for the upper shore which is estimated using the ecotype that is native to that habitat (Appendix S3).

## 3 Results

### 3.1 Phenotypic divergence between ecotypes

The phenotypic characterization showed that the two ecotypes differed significantly in shell and life‐history traits (Table 1). When shell weight was plotted as a function of shell length (using only snails of shell length 3–5 mm), no significant difference in the allocation of shell material as a function of shell length between ecotypes was observed (paired *t* test *p *=* *.62, Fig. S4a,b). The dry body weight of the wave ecotype increased more steeply with shell length than the body weight of the crab ecotype (Fig. S4c). The wave ecotype had a significantly thinner shell than the crab ecotype (Fig. S4d; Table 1). The geometric morphometrics analysis showed a significant difference in shell aperture shape (RW1) between the crab ecotype and the wave ecotype (Fig. S4e). Differences between the two ecotypes were also observed for the geometric morphometrics variables U1 (*p* < .000), RW1 (*p* < .000), and RW4 (*p* < .001) (data not shown). Finally, to facilitate comparison with the other traits, RW1 was plotted against shell length (Fig. S4). Fortunately, shell length was highly correlated with centroid size (Fig. S4f). In contrast, when dry shell weight was plotted as a function of dry body weight (using only snails of body weight less or equal to 11.2 mg), a significantly steeper slope was observed for the crab ecotype than for the wave ecotype (*t* test for equality of slopes *p *<* *.001, Fig. S5), showing the higher allocation of shell weight per gram of body weight by the crab ecotype relative to the wave ecotype.

The most notable difference between the two ecotypes was in the delayed sexual maturity of the crab ecotype. All females and all but one male of the wave ecotype were sexually mature at the minimum shell length of 3.0 mm (Fig. S4a,b). In contrast, the smallest mature male of the crab ecotype had a shell length of 8.1 mm, while the smallest female had a shell length of 7.2 mm (Fig. S4a,b). A Fisher's exact test of association comparing the number of mature females and the number of juveniles for the two ecotypes within the size range was highly significant (*n* = 92, *df*^**^ *=** ***1, *p *<* *.001). A Fisher's exact test of association comparing the number of mature males and the number of juveniles/immature males for the two ecotypes was also highly significant (*n* = 83, *df* = 1, *p *<* *.001).

### 3.2 Laboratory predation experiments

#### 3.2.1 4‐mm and 9‐mm crab ecotype predation experiment

**Figure 2:**
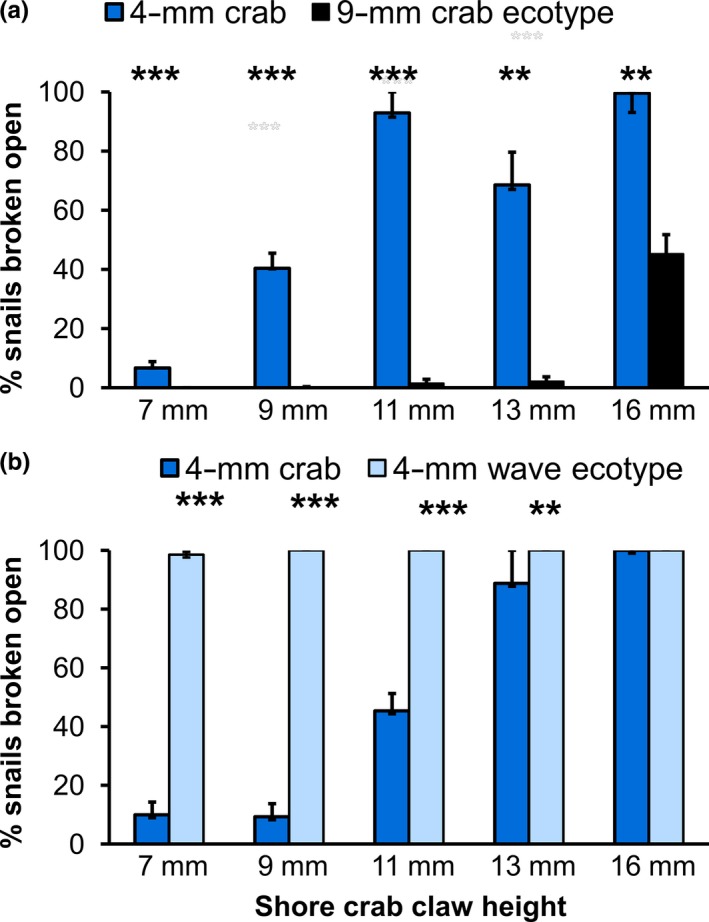
Laboratory consumption rate per crab per trial for each claw height size‐class (mean and standard error) when offered 10 prey of each of two categories. (a) First laboratory experiment using 4‐mm crab and 9‐mm crab ecotypes, *n* = 7 trials × 20 crabs; Table S2). (b) Second laboratory experiment 4‐mm crab and 4‐mm wave ecotypes (*n* = 4 trials × 19 crabs; Table S4). Fisher's exact test: *p* < .001*** *p* < .01**

The different size‐classes of crabs differed dramatically in their consumption of the 4‐mm and the 9‐mm prey categories of the crab ecotype. The smallest size class (7 mm CH) of the marbled shore crab ate almost none of either prey category (Figure 2a). The intermediate sizes of crabs (9 mm, 11 mm, and 13 mm CH) consumed the 4‐mm prey category at a moderate rate but ate almost none of the 9‐mm prey category. Only the extremely large (16 mm CH) size‐class of crabs had a high consumption rate of the 9‐mm prey category in addition to the 4‐mm prey category (Figure 2a).

The importance of CH was supported by a logistic regression analysis that showed a significant interaction between prey category and CH, thus showing that the largest crabs (16 mm CH) ate significantly more snails in the 9‐mm prey category than did the smaller crabs (Table S2). Adding a CH squared term to the model did not improve the model fit but adding the interaction between CH and snail size‐class did improve the fit (Table S2).

**Figure 3:**
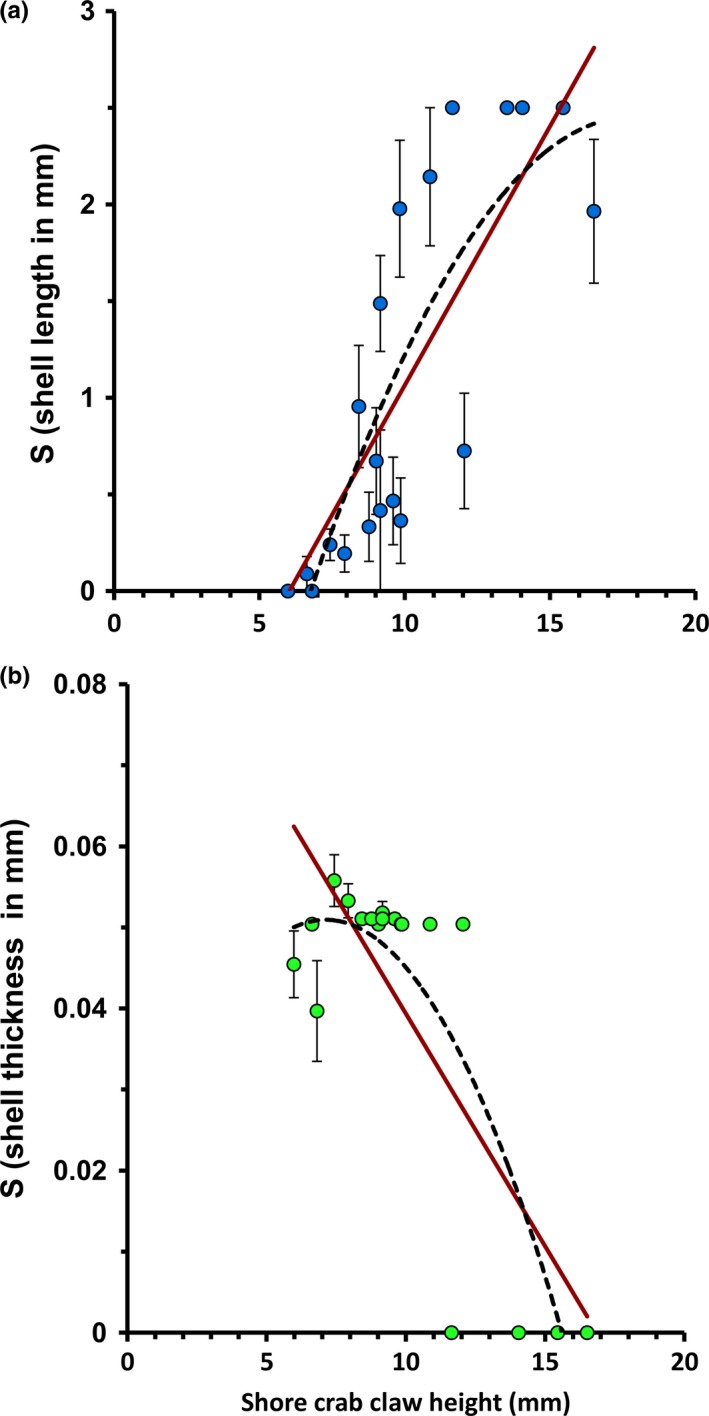
Mean (±*SE*) univariate linear selection differential for each individual crab in the laboratory experiments plotted against the crab's claw height. (a) First laboratory experiment using 4‐mm crab and 9‐mm crab ecotypes (*n* = 7 trials × 20 crabs; Table S3). (b) Second laboratory experiment using 4‐mm crab and 4 mm wave ecotypes (*n* = 4 trials × 19 crabs; Table S5). Linear and quadratic polynomial regression curves are shown

Linear selection differentials for shell length significantly increased with CH (Table S3; Figure 3a) and adding a quadratic term to the model (AIC_c_ = 42.042) that did not result in a significantly better fit than a linear regression (AIC_c_ = 40.895; Figure 3a). With one exception, the strongest directional selection on shell length was provided by the largest crabs used in the laboratory experiment, which were less selective but consumed more snails per trial than did the medium‐sized crabs.

All possible subsets multiple regression showed that the set of crab measurements that best predicted the selection differential for the crab ecotype contained CH alone (AIC_c_ = −103.883), which did not fit significantly better than claw length alone (AIC_c_ = −103.341), but did fit better than the combination of claw length with CW AIC_c_ = −100.850).

#### 3.2.2 4‐mm crab ecotype and wave ecotype predation experiment

All five size classes of crabs were similar in eating 100% of the wave ecotype prey category (Figure 2b), but they differed considerably in their consumption of the crab ecotype prey category. It was notable that when individuals of the wave ecotype were available as alternative prey, the medium (9 mm CH) size class of crabs consumed an average of only one crab ecotype individual per trial (Figure 2b), instead of the five crab ecotype individuals per trial that the same crabs had consumed when the 9‐mm crab ecotype was the only alternative prey. This was supported by logistic regression analysis, which found that a model with the square of CH and with a significant interaction between ecotype and individual crab CH fit significantly better than alternative models (Table S4). The handling time of the wave ecotype individuals was observed to be very short. Most crabs broke all the wave ecotype snails open within the first hour while the crab ecotype snails remained alive for several hours or days (E. G. Boulding, personal observation). In contrast to the previous experiment, the selection differential decreased significantly with crab CH (Figure 3b, Table S5; *p *=* *.0025). The selection differentials for shell thickness as a function of crab CH fit a quadratic polynomial regression (AIC_c_ = −763.498) better than they fit a linear regression (AIC_c_ = −747.481; Figure 3b); the left part of the curve is missing, but suggests that very small crabs with CH s smaller than 5 mm would exert smaller selection differentials on shell thickness. The effect of dactyl length was supported by our observation that our smallest crab attempted to “winkle” out the body of wave ecotype snails by putting its entire dactyl into the shell aperture (https://www.youtube.com/watch?v=QSCvd9gYjk0&feature=em-upload_owner). Shells from which snails had been removed by small crabs using the winkling technique sometimes had a small hole punched in the wall of the last body whorl.

All possible subsets multiple regression showed that the crab measurements that best predicted the selection differential that the crabs exerted on the snails was a combination of CH and dactyl length (AIC_c_ = 62.343), which fit similarly to a model that considered CH alone (AIC_c_ = 63.040), but better than models with only claw length (AIC_c_ = 65.834) or CW and CH (AIC_c_ = 65.834). Therefore, the model with CH alone was again used.

### 3.3 Tethering experiments

#### 3.3.1 4‐mm and 9‐mm crab ecotype tethering experiment

**Figure 4:**
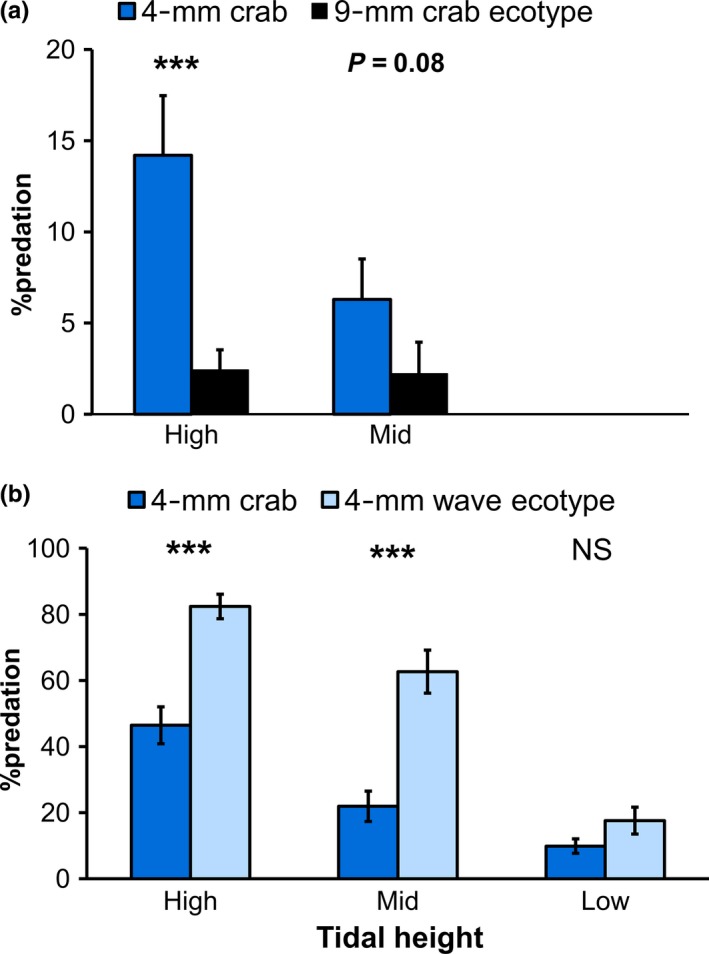
Effect of tidal height on the percentage of tethered snails classified as being eaten by a marbled shore crab. (a) First field experiment using 4‐mm crab and 9‐mm crab ecotypes (Table S6). (b) Second field experiment using 4‐mm crab and 4 mm wave ecotypes (Table S7)

Analysis of the first tethering experiment—assuming that the tethers with attached shell fragments and the “epoxy‐only” category both represented valid predation events—showed that the large prey category of the crab ecotype survived better on the upper shore than did the small prey category. On the upper shore (High), there is a clear trend showing higher predation on the 4‐mm prey category than on the 9‐mm prey category (Figure 4a). Size × fate was included in the most parsimonious log‐likelihood model (Table S6). A likelihood ratio chi‐square test of association of size‐class and fate was highly significant (Table S6). The predation rate at the Mid level at transect 1 was like that at the High level. However, the Mid level of transect 3 showed very low predation on tethered snails while transect 2 Mid did not show predation at all. The observation of predation at the Mid level of transect 1 may explain why likelihood ratio chi‐square test for association of transect and fate was significant, but the association of tidal level and fate was not (Table S6).

Reanalysis of this first tethering experiment—counting only tethers with attached shell fragments as valid predation events—gave very similar results with the large crab ecotype surviving significantly better than the small crab ecotype (Table S6). Comparison of the tethered shell fragments with those produced in our laboratory predation experiments strongly suggested crab predation. Predation rates were highest near cracks of sufficient size to offer refuge to marbled shore crabs during low tide (Fig. S1b, Fig. S6).

#### 3.3.2 4‐mm crab ecotype and wave ecotype tethering experiment

Analysis of the second tethering experiment—assuming that the tethers with attached shell fragments and the “epoxy‐only” tethers both represented valid predation events—showed a significantly higher survival of the 4‐mm crab ecotype compared with the 4‐mm wave ecotype at the High level of all three transects and also at the Mid level of transect 1, with very little predation by crabs occurring at the Low level (Figure 4b). The likelihood ratio chi‐square test revealed an association between ecotype and predation mortality and between tidal level and predation mortality. There was no significant association between transect and predation mortality (Table S7).

Reanalysis of this second tethering experiment—counting only tethers with attached shell fragments as valid predation events—gave very similar results with the small 4‐mm crab ecotype surviving significantly better than the small 4‐mm wave ecotype. Likelihood ratio chi‐square tests produced similar results, except the association of transect and predation mortality was no longer significant (Table S7).

#### 3.3.3 Univariate linear selection differentials and gradients from the field tethering

The univariate standardized selection differential, i, and the standardized linear selection gradient, β1, for shell length from tethering experiment 1, which used the 4‐mm and 9‐mm crab ecotypes prey categories (Table 2), were positive and significant for the High transects 1 and 2. They were also positive and significant for tethering experiment 2, which used the wave and crab ecotypes of the 4‐mm size class for the High transects 1, 2, and 3 (Table 3). Most interestingly, the standardized linear selection gradients were smaller for the first experiment with shell length than for the second experiment with shell thickness (Tables 2 and 3).

**Table 2:** Mean selection differential, *S*, and mean standardized selection differential, *i*, from four trials (*N*_S_) of tethering experiment 1. The univariate linear selection gradient, β_1_, for shell length was estimated using the pooled data from all four trials

Transect	Level	*N*_S_	*S*(mm) ± *SE*	*i* ± *SE*	*N*_β1_	β_1_ ± *SE*	*p*_β1_
1	High	4	0.196 ± 0.0893	0.0793 ± 0.0361	119	0.0800 ± 0.0315	**.012** b
1	Mid	4	0.143 ± 0.0520^a^	0.0578 ± 0.0210^a^	117	0.0577 ± 0.0356	.107
2	High	4	0.276 ± 0.0833^a^	0.112 ± 0.0336^a^	114	0.114 ± 0.0358	**.002** b
2	Mid	4	0.000 ± 0.000	0.000 ± 0.000	117	0	–
3	High	4	0.0460 ± 0.0460	0.0186 ± 0.0186	119	0.0178 ± 0.0126	.159
3	Mid	4	0.0217 ± 0.0221	0.00877 ± 0.00877	116	0.00908 ± 0.00889	.309

a 95% confidence limits on the mean do not overlap zero.

b Bold font indicates slope is significantly different from zero.

**Table 3:** Mean selection differential, *S*, and mean standardized selection differential, *i*, from seven trials (*N*_S_) of tethering experiment 2. The univariate linear selection gradient, β_1_, for shell length was estimated using the pooled data from all seven trials

Transect	Tide_level	*N*_S_	*S*(mm) ± *SE*	*i* ± *SE*	*N*_β1_	β_1_ ± *SE*	*p*_ß1_
1	High	6	0.0151 ± 0.00874	0.305 ± 0.176	115	0.235 ± 0.091	**.011** b
1	Mid	7	0.0377 ± 0.00782^a^	0.754 ± 0.155^a^	103	0.616 ± 0.078	**.000** b
1	Low	7	0.00349 ± 0.00138	0.0752 ± 0.0300^a^	94	0.148 ± 0.105	.160
2	High	7	0.0261 ± 0.00755^a^	0.520 ± 0.150^a^	116	0.353 ± 0.088	**.000** b
2	Mid	7	0.0112 ± 0.00412^a^	0.225 ± 0.0827^a^	112	0.311 ± 0.092	**.001** b
2	Low	7	0.000713 ± 0.00131	0.0141 ± 0.0264	103	0.063 ± 0.101	.537
3	High	7	0.0326 ± 0.00576^a^	0.650 ± 0.115^a^	122	0.545 ± 0.077	**.000** b
3	Mid	7	0.0168 ± 0.00439^a^	0.341 ± 0.0909^a^	117	0.378 ± 0.087	**.000** b
3	Low	7	0.00314 ± 0.00243	0.0626 ± 0.0485	96	0.143 ± 0.102	.163

a Significant because the 95% confidence limit on the mean does not overlap 0.

b Bold font indicates slope is significantly different from zero.

#### 3.3.4 Estimation of the width of the predatory crab zone

The transect‐specific results from the two tethering experiments (Figure 1c) showed that the width of the upper intertidal zone where predation by marbled shore crabs was taking place was <38 m at transect 1, 26.2 m at transect 2, and 18.5 m at transect 3. We confirmed the presence of the marbled shore crab in this zone by extensive field observations and by placing frozen fish as bait in tide pools to lure them from their crevices. We observed no other crab species capable of breaking open the tethered snails at this site.

### 3.4 Theoretical versus observed scales of local adaptation

#### 3.4.1 Characteristic length using estimates of ω from tethering experiments

Nonlinear regression estimated that the standard deviation of a Gaussian fitness function was much greater for shell length than for shell thickness, as would be expected if stabilizing selection was stronger for shell thickness than for size (Table 4, Fig. S7a). An estimate of ω = 2.72 (95% CL: 2.190–3.254) was obtained using binary survival for the High level of the first field tethering experiment with the 4‐mm and 9‐mm size class of tethered crab ecotype which gave a characteristic length, *L*_c_ = 22.2 m (Table 4). Similarly, an estimate of ω = 0.549 (95% CL: 0.442–0.656) was obtained using the binary survival for the High level of the second field tethering experiment with the 4‐mm crab ecotype and wave ecotype (Table 4) which gave *L*_c_ = 8.75 m (Table 4).

**Table 4:** Characteristic length, *L*_c_ (Slatkin, 1978), from parameters estimated here and those from a previous field study on another direct developing species of *Littorina* and the estimate (95% confidence limits) and values of the parameters used to calculate *L*_c_

Species (shell length)	Trait	*SD* a	∆θ	ω	*d*	*h* ^2^	*L*_c_
*L. saxatilis* 4‐mm crab vs. 9‐mm crab	Shell length, mm	1.12	−1.38	H: 2.72 (2.19 −3.25)	4.2^b^ (0.84^c^–5.6^d^) m^e^	0.3	22.2 (4.46–29.6) m
*L. saxatilis* 4‐mm crab vs. 4‐mm wave	Shell thickness, mm	0.359	−0.279	H: 0.549 (0.442**–**0.656)	4.2^b^ (0.84^c^–5.6^d^) m^e^	0.3	8.75 (1.76–11.7) m
*L. subrotundata* vs. *L. sitkana* medium^f^	Shell weight, mg	3.2	−2.44	2.60	5.8 m	0.3	29.5 m
*L. subrotundata* vs. *L. sitkana* large^f^	Shell weight, mg	3.2	−3.65	1.71	5.8 m	0.3	21.0 m

a *SD* is one standard deviation for the trait (Table 1), which was used to express the model parameters Δθ and ω in PSD = phenotypic standard deviation units.

b Median vertical migration rates when transplanted back to their native tidal height = 1.5 m/month (Erlandsson et al., 1998).

c Median vertical migration rates from controls back to the same tidal level maximum 0.3 m/week (Cruz et al., 2004b).

d Median vertical migration rates for the “crab” ecotype transplanted back to the high shore at a different site was estimated at 2 m/month (Erlandsson et al., 1998).

e Median migration rate were multiplied by 2.8 to convert to dispersal distance, *d* (Wright, 1969).

f From Table 1 in Boulding et al. (2007).

#### 3.4.2 Spatial scale of observed divergence

Divergence among populations of the two ecotypes was highly significant for shell length, shell weight, body weight, shell thickness, and aperture shape (RW1) when a distance between pure populations of each ecotype of 20 m was assumed (Table 1). The standardized spatial scales of divergence and adaptation were between 0.36 and 1.0 Wrights when the maximum median lifetime dispersal distance of 4.2 m was used (Table 1).

## 4 Discussion

Our field tethering and laboratory results clearly show that the small size, thin shell, and large shell aperture of the wave ecotype result in it being poorly adapted to high shore regions where the marbled shore crab is abundant. We have increased the understanding of predator‐driven divergent selection within this model system by demonstrating (1) strong natural selection for increased shell size of the crab ecotype on the high shore, (2) nonlinear changes in the selection differentials on shell size and on thickness with increasing gape size of the claw of the marbled shore crab, (3) enemy‐free refuges for the wave ecotype in the low intertidal zone, (4) predator‐driven immigrant inviability for the wave ecotype tethered on the high shore, and (5) predicted characteristic lengths of microgeographic adaptation from our parameterization of Slatkin's (1978) model showing that the physical distances between the upper and lower shore habitats are sufficient to result in ecotype formation. These results are discussed sequentially below:

### 4.1 Support for size being a magic trait in *Littorina saxatilis*

Our data showing spatially differential selection for larger shell size support our hypothesis that size represents a classic magic trait (Servedio et al., 2011) with respect to the evolution of crab and wave ecotypes. We found significant and strong directional selection for larger size and for increased shell thickness at the High tidal level but did not observe it at the Mid or Low intertidal levels. The large differences in size at sexual maturity between the two ecotypes (size at sexual maturity <3.5 mm for the wave ecotype and >8 mm for the crab ecotype, Fig. S4a,b; see also Table 6 from Johannesson et al., 1995), in combination with the size‐assortative mating detected in previous studies (reviewed in Rolán‐Alvarez, 2007), may cause a larger than expected reduction in hybridization as a by‐product, because crab ecotype individuals below 8 mm will be immature whereas few wave ecotype individuals exceed 6 mm.

The benefit‐to‐cost ratio of an early maturing strategy to a late maturing strategy can change with the presence or absence of a gape‐limited predator (Urban, 1997); predator‐prey coevolution can theoretically result in two coexisting prey species (Day, Abrams & Chase, 2002). However, local coevolution is unlikely for the marbled shore crab because of its prolonged free‐swimming larval phase (Silva, Mesquita, Schubart, Alves & Paula, 2009). We present strong evidence that the upper limit of gape size of its principal predator was the cause of this spatially differential selection for the large size and delayed maturity of the crab ecotype. We showed that the 9‐mm size‐class of the crab ecotype had a size refuge from marbled shore crabs with a CH of <14 mm (Figure 2a). Only male crabs with CW larger than 33 mm have claws this large, and these represent a small proportion of the population at this site (E. G. Boulding, personal observation). Previous work on other *Littorina* species has also shown that larger individuals or crab species with larger claw gapes can consume individuals of a thicker‐shelled ecotype (or species) at a higher rate because they can crush them outright within their claw rather than by the more time‐consuming outer‐lip peeling technique that must be used then they are too large to within the claw gape (Behrens Yamada & Boulding, 1998; Johannesson, 1986).

Even if collecting 9‐mm size‐class of the wave ecotype had been possible at this site, the literature predicts that we would not have observed selection favoring larger body size on the high shore for the wave ecotype. Previous predation experiments with another shore crab (*Hemigrapsus nudus*) and another thin‐shelled species of *Littorina* found that when prey was not limiting, increasing the prey size from small to medium size‐class increased the ratio of the preference for the thin‐shelled species (*Littorina subrotundata*) over the thick‐shelled species (*L. sitkana*) from 1.74 to 18.3 (Boulding et al., 2007 Supplement page 18). This likely reflects increasing interspecific differences in the positive allometry of shell thickness with increasing size (Boulding et al., 2007).

We did not estimate the magnitude of selection against the larger size or thicker shells in the lower shore. Reviewing the literature (Rolán‐Alvarez, 2007; Rolán‐Alvarez et al., 2015) makes us doubt the wave ecotype experiences direct selection for thinner shells on the low shore. The wave ecotype could experience indirect selection for thinner shells on the lower shore if the maximum rate at which shell material can be produced limits the rate of body growth (Palmer, 1981) of the wave ecotype.

### 4.2 Nonlinear changes in selection differentials with predator gape size

We showed that selection differentials on prey size changed with changes in shore crab claw gape. In our first laboratory experiment, the preference for the small versus the large crab ecotype prey category, and therefore directional selection, increased significantly with crab CH (Figure 4a). This was in part because crabs with CHs smaller than 8 mm ate none of either size‐class of crab ecotype. In contrast, our second laboratory experiment clearly showed that the strongest directional selection for increased shell thickness was provided by the intermediate‐sized crabs with intermediate‐sized gapes rather than by the larger crabs (Figure 4b). Decrease in the selection differentials for shell weight with increasing crab size has been shown for a trait correlated with shell thickness in another *Littorina* species (Pakes & Boulding, 2010).

A limitation of our laboratory predation experiments with the 4‐mm crab and wave ecotypes was that we only used two phenotypic categories of snails that are known to differ not only in shell thickness but also in aperture shape (Rolán‐Alvarez, 2007; Fig. S4e). Future work to find direct and indirect targets of selection could measure all potentially important traits that can be measured on live snails before deployment so that multivariate selection gradients could be estimated (Lande & Arnold, 1983; Mitchell‐Olds & Shaw, 1987). These gradients in conjunction with estimates of the genetic variance–covariance matrix (Boulding & Hay, 1993) would allow prediction of the multivariate response to selection. This would be a massive undertaking. For our second laboratory experiment with 20 crabs, it would have been necessary to measure 400 individual snails for each of the four trials.

### 4.3 Predator‐free space in the low intertidal zone

The second tethering experiment showed that shell‐breaking predation by the marbled shore crab was very rare at our low tidal level, providing the wave ecotype with “enemy‐free space” (sensu Jeffries & Lawton, 1984) on the lower shore. This spatial escape from predation may have permitted the wave ecotype to evolve a small size at maturity, and a flat shell shape, to exploit either small cracks (Emson & Faller‐Fritsch, 1976) or small biogenic refuges from heavy surf (Rickards & Boulding, 2015) created by mussel beds. Where small size in the lower shore is favored, that should concomitantly favor sexual maturity at smaller sizes by paedomorphosis (Diz, Páez de la Cadena, & Rolán‐Alvarez, 2012). The wave ecotype also has a large aperture which permits a larger foot and reduces dislodgment (Trussell, Johnson, Rudolph, & Gilfillan, 1993). Large individuals of the wave ecotype might be dislodged in storms once they grew too large to retreat deeply inside the small biogenic refuges (Boulding & Van Alstyne, 1993) which here are between mussel clumps (E. G. Boulding, personal observation). This requirement of the wave ecotype to fit into small biogenic refuges on the lower shore and the lower number of shore crabs observed there has previously been hypothesized to result in divergent selection on size between the upper and lower shores (Johannesson, Rolán‐Alvarez, & Erlandsson, 1997). Wave ecotype snails (>4 mm SL) have been shown to grow more slowly than juvenile crab ecotype snails of similar size even when monitored in their “home” habitats (Johannesson et al., 1997), however no field growth data exist for juveniles of the wave ecotype.

### 4.4 Predator‐driven immigrant inviability in the high intertidal zone

Recent work has focused on how predator‐driven immigrant inviability can help to drive trait divergence and incipient reproductive isolation by causing the spatial separation of ecotypes (Ingley & Johnson, 2016; Nosil, 2012; Nosil & Crespi, 2004; Nosil et al., 2005; Schluter, 2000). The results of our tethering experiments were broadly like those of extensive reciprocal transplant experiments in which the wave ecotype survived poorly on the upper shore (Cruz et al., 2004a; Rolán‐Alvarez et al., 1997). Although divergent selection has been inferred from previous field experiments and genetic studies (reviewed in Rolán‐Alvarez et al., 2015), the role of immigrant inviability through crab predation as a barrier to gene flow has not previously been evaluated in these populations. The shell fragments recovered in both tethering experiments showed that there exists a high‐risk predator zone in the splash zone just along the upper limit of the barnacle zone. The marbled shore crab has a small “home range” (Cannicci, Paula, & Vannini, 1999). If this range includes abundant refuges from its own predators, then it may result in the higher predation rates that we observed for snails tethered near cracks.

Previous shell morphometric studies along this cline have suggested that divergent selection acts on the ratio of shell aperture size to body size. A larger aperture allows a larger foot which is useful in the surf zone on the lower shore (Carvajal‐Rodriguez et al., 2005; Conde‐Padin et al., 2007). Our observations of the marbled shore crabs preying on the wave ecotype suggested that aperture size also affects vulnerability to the smallest crabs. We observed in the 4‐mm wave ecotype that a larger aperture allowed very small shore crabs (CH 7 mm) to insert the movable finger of their claws into the shell or to use both fingers as forceps to remove chunks of the snail's body.

### 4.5 Theoretical versus observed scales of local adaptation

This study presents the first characterization of significant microgeographic divergence (sensu Richardson et al., 2014) in shell thickness, shell weight, body weight, aperture shape, and size at sexual maturity for crab and wave ecotypes of *L. saxatilis* from NW Spain.

These patterns of empirical divergence can be compared with the characteristic length predicted by our parameterization of Slatkin's (1978) model of a cline in a quantitative trait. Relative fitness estimates from the first tethering experiment with 4‐mm and 9‐mm size classes of the crab ecotype resulted in a characteristic length of 22 m for the trait size (Table 4). The width of the intertidal zone (length along the beach) inhabited by the marbled shore crab was greater (28 m on average; range, 18–38 m [Figure 1c]), suggesting that local adaptation to the crabs should occur. Similarly, relative fitness estimates from our 4‐mm crab ecotype and wave ecotype tethering experiment gave us an estimate of the characteristic length of 8.4 m (Table 4). Again, the width of the intertidal zone inhabited by the marbled shore crab was much greater than this and exceeded 28 m (Figure 1c). Previous simulations using parameters from this system also support vertical microgeographic differentiation being possible in this system (Pérez‐Figueroa, Cruz, Carvajal‐Rodriguez, Rolán‐Alvarez, & Caballero, 2005). Characteristic lengths of the same order of magnitude were found for a wave‐adapted species (*L. subrotundata*) in a northeastern Pacific field experiment that simulated a predatory crab invasion (Table 4).

Spatial extent and position of the width of the intertidal zone containing predatory crabs may be critical in permitting ecotype formation. On the almost atidal west coast of Sweden, the wave and crab ecotypes of *L. saxatilis* are not found along a vertical intertidal gradient, but instead are distributed along a horizontal gradient of wave exposure (Galindo & Grahame, 2014; Janson, 1983). Based on the parameters and calculations presented here, we would argue that the minimum width of the ecotone required for a crab ecotype to evolve in the lower part of a wave‐exposed shore in Sweden would be much larger than the intertidal range. Our hypothesis is supported by estimates of the average width of horizontal clines for single AFLP loci between the crab and wave ecotypes of Swedish *L. saxatilis* ranging from 10.4 to 23.8 m (Hollander, Galindo, & Butlin, 2015) being too long to fit vertically within an atidal shore.

### 4.6 Conclusions and implications for incipient speciation

Our work adds to previous studies showing strong predator‐mediated selection within only a portion of the microgeographic distribution of a prey species could drive local ecotype formation. This hypothesis is supported by the similarity between theoretical versus observed scales of microgeographic adaptation by the prey, suggesting that extrinsic predator‐mediated mechanisms are sufficient even for nonmagic traits such as shell thickness. The predator‐free space created by the abiotic constraints on the lower shore permits the poorly dispersing prey species to evolve a small, flat shell with a large aperture that may reduce dislodgement in heavy surf. Yet this adaptation to the predator‐free space on the lower shore results in predator‐driven immigrant inviability on the upper shore where the marbled shore crab is abundant. We show that adults of the thick‐shelled crab ecotype have evolved a size refuge from gape‐limited predation by most marbled shore crabs. The existence of the size refuge from predation would result in direct selection for rapid juvenile growth and indirect selection for late maturity. Our results support the action of strong divergent selection for shell size at maturity in thick‐shelled individuals of this species and size is also involved in the evolution of reproductive barriers through sexual selection (assortative mating) (Rolán‐Alvarez, 2007; Rolán‐Alvarez et al., 2015). This in conjunction with the size‐assortative mating described in previous studies (reviewed by Rolán‐Alvarez et al., 2015) supports the hypothesis that shell size represents a magic trait in this system.

Our parameterization of Slatkin's (1978) model suggests that directional selection by the marbled shore crab is spatially restricted and strong enough to permit large shell size and thick shells to evolve on the upper shore despite putative opposing directional selection for small size and early maturity on the lower shore. Our second set of laboratory and tethering experiments showed that even at a small size possession of a thick shell with a small aperture resulted in substantially higher fitness in the presence of the marbled shore crab. Our first set of laboratory and tethering experiments shows that directional selection on thick‐shelled ecotype for larger shell size on the upper shore. We hypothesize that size is a magic trait such that an increase in size at sexual maturity would increase assortative mating which in turn would facilitate the rapid diversification of the two ecotypes. Shell thickness is unlikely to be a magic trait in this system, yet Slatkin's (1978) model suggests that conditions are present such divergence in size thickness between the upper shore areas inhabited by crabs and the lower shore predator‐free refuge could still occur. We agree that molecular work has been helpful (Butlin et al., 2014) but not sufficient (Travisano & Shaw, 2013) to resolve still poorly understood aspects of this experimentally tractable model system. Future work should estimate the magnitude and targets of selection on the wave ecotype living in the low shore as well as increase our understanding of which traits influence assortative mating in the middle shore.

## Data archiving

Raw data used in our analyses will be archived under dryad. Data available from the Dryad Digital Repository: http://dx.doi.org/10.5061/dryad.jb10h

## Conflict of Interest

None declared.
