# Supplementary material for: Size selection by a gape‐limited predator of a marine snail: Insights into magic traits for speciation
Source: Ecol Evol. 2016 Dec 20;7(2):674–88. doi: 10.1002/ece3.2659 (PMC5243190; doi:10.1002/ece3.2659)
Supplement: Supplementary file 1 [file ECE3-7-674-s001.pdf]

APPENDIX 1 DETAILED METHODS LABORATORY PREDATION EXPERIMENTS

SAMPLING AND ANIMAL MAINTENANCE

Littorina saxatilis was sampled at random from two microhabitats (5–7 m^2) below the Cabo Sillero lighthouse in near Baiona, Spain:

https://www.google.ca/maps/@42.1033578,-8.8969041,383m/data=!3m1!1e3

The crab ecotype snails were collected on the sheltered side of a large stack to the right of T3 HIGH whereas wave ecotype snails were mostly collected from between T2 LOW and T3 LOW (Fig. 1). Snails were then taken to the laboratory at ECIMAT marine station (Estación de Ciencias Mariñas de Toralla (ECIMAT), University of Vigo, Galicia, Spain; 42°12′02″N; 8°48′00″W). There they were maintained in glass aquaria (18 x 10 x 6 cm) with free-flowing sea water and following the conditions (temperature, aeration, feeding, etc.) described by Saura *et al*. (2011). Samples maintained in the laboratory for longer than 15 days were not included in the experiments.

Samples of the marbled shore crab (*Pachygrapsus marmoratus*) (Fig. 1A) were collected at Canto da Area beach (42°10′56″N; 8°48′43″W; Galicia, Spain) on April 4, 2014 and also taken to the laboratory at ECIMAT. Each individual crab was maintained in an individual glass aquarium (10.5 x 11 x 6 cm) with open circuit of sea water and with the same conditions as described above for the snails (Fig. S1B). The aquaria containing crabs of the small size class (15–19 mm) were provided with an overturned petri dish (100 mm diameter x 20 mm high) in the bottom of the aquarium to ensure that the crab could reach any snails that crawled onto the aquarium roof (underside of the glass cover). The crabs were fed with snails of different sizes of the crab ecotype until the experiment began. Three crabs molted during the experiments; these

were held until their carapaces hardened, remeasured, and then included in the experiments again.

Snails recovered from each aquarium were classified into the following categories: AU (Alive Undamaged), AC (Alive Chipped), AP (Alive Peeled), DP (Dead Peeled), DU (Dead Undamaged), DC (Dead Chipped), DF (Dead with shell Fragment, and M (Missing). For the main statistical analysis AU, AC, AF, and AP were coded as “1” for not killed, DF, DP, and DC were coded as “0” for likely to have been killed by crabs, and DU and M were coded as missing data.

A third laboratory predation experiment at ECIMAT showed that very large (claw height=13 mm) and extra-large (claw height=16 mm) marbled shore crabs pulled the snail off the epoxy used to attach the snail’s shell apex to the tethering line more than 50% of the time. After consumption of two of 4-mm crab ecotype and two wave ecotype by each of three crabs, the recovered tethering lines were classified as six Epoxy Only, five DF, and one Knot Only (Appendix 3).

SELECTION GRADIENTS FROM LEAST SQUARES REGRESSION

We decided to present our univariate selection gradients using standardized linear least squares regression coefficients (Lande and Arnold 1983) as these can more easily be used for meta-analyses of selection (Kingsolver and Diamond 2011; Siepielski *et al.* 2013). Before estimation the independent prey trait variable was standardized by its mean and standard deviation, while the dependent binary survival values were transformed by dividing by the average survival of both prey categories for a particular replicate of a particular experiment. Linear least squares regression analyses were then carried out using the “by groups” option in

Systat. There are known problems with using least squares regression to estimate univariate selection gradients, including the possibility of there being a correlated independent variable that is causal with respect to fitness that one did not include in the model (Mitchell-Olds and Shaw 1987) and statistical problems with using a binary fitness variable as an independent variable (Janzen and Stern 1998). Therefore, we also used logistic regression models to determine the statistical significance of the laboratory predation experiments.

References only cited in Appendix 1:

Barbeau, M. A., and R. E. Scheibling. 1994. Procedural effects of prey tethering experiments: predation of juvenile scallops by crabs and sea stars. Mar. Ecol. Prog. Ser. 111:305–310.

Cannicci, S., M. Gomei, B. Boddi, and M. Vannini. 2002. Feeding habits and natural diet of the intertidal crab *Pachygrapsus marmoratus*: Opportunistic browser or selective feeder? Estuarine Coastal Shelf Sci. 54:983–1001.

Flores, A. A. V., and J. Paula. 2002. Population dynamics of the shore crab *Pachygrapsus marmoratus* (Brachyura: Grapsidae) in the central Portuguese coast. J. Mar. Biol. Assoc. U. K. 82:229–241.

Janzen F. J., and H. S. Stern. 1998. Logistic regression for empirical studies of multivariate selection. Evolution 52:1564-1571.

Kingsolver, J. G., and S. E. Diamond. 2011. Phenotypic selection in natural populations: what limits directional selection? Am. Nat. 177:346–357.

Siepielski, A. M., K. M. Gotanda, M. B. Morrissey, S. E. Diamond, J. D. DiBattista, and S. M. Carlson. 2013. The spatial patterns of directional phenotypic selection. Ecol. Lett. 16:1382–1392.

Silva, A., D. Boaventura, A. Flores, P. Re, and S. J. Hawkins. 2004. Rare predation by the intertidal crab *Pachygrapsus marmoratus* on the limpet *Patella depressa*. J. Mar. Biol. Ass. U. K. 84:367–370.
