# Supplementary material for: Size selection by a gape‐limited predator of a marine snail: Insights into magic traits for speciation
Source: Ecol Evol. 2016 Dec 20;7(2):674–88. doi: 10.1002/ece3.2659 (PMC5243190; doi:10.1002/ece3.2659)
Supplement: Supplementary file 2 [file ECE3-7-674-s002.pdf]

APPENDIX 2. EXTENDED METHODS DIVERGENCE BETWEEN ECOTYPES

We quantified the differences in shell, body, and life history traits between ecotypes across a broad size range of those available at Cabo Silleiro. We randomly chose 10 snails within each 0.5 mm interval from 3–13 mm in shell length for the “crab” ecotype and from 3–6 mm for the “wave” ecotype from the *Littorina saxatilis* that had been maintained in the laboratory for less than 15 day. We then boiled each snail for approximately 5–10 s, removed its entire body from its shell using forceps, and recorded its developmental stage and, when possible, its gender (juvenile, mature female containing embryos, immature male with bump at future location of penis, mature male with fully developed penis). The body and shell were dried separately at 60 ºC for 72 h in a drying oven (FD-53, Binder GmbH, Tuttlingen, Germany) and their dry weights were measured with an analytical balance (LE225D, Sartorius, AG, Göttingen, Germany). The shells were digitally photographed (Fig. S1d-e) and digitizing was performed using 11 landmarks used previously for these ecotypes (Carvajal-Rodríguez *et al.* 2005, Conde-Padín *et al.* 2009). Geometric morphometric analysis of the landmarks used the free programs tpsUtil, tpsDig and tpsRelw available at http://life.bio.sunysb.edu/morph/ (Rohlf 2015). The geometric morphometric trait values: centroid size (CS), two uniform components (U1, U2), and 10 relative warps (RW1, RW2, RW3, RW4, RW5, RW6, RW7, RW8, RW9, RW10) were obtained for each snail. Of these U2, RW3, RW5, RW6, RW7, RW8, RW9, RW10 did not differ significantly among ecotypes (t-test assuming unequal variances α=0.01) so are not considered further.

Shell thickness of the outer shell lip was measured parallel to the columella axis (three replicate measurements) using a modified digital metric dial gauge (0.001 mm) with a magnetic base stand (Fig. S2). A digital metric dial gauge (0.001 mm precision) was attached to a magnetic base stand, which allowed vertical movement of the dial gauge. The magnetic base stand was

fixed to a heavy iron stand to stabilize the gauge. Prior to beginning each measurement, the hypodermic needle that was attached to the dial gauge by the adaptor was moved so that its distal end contacted the top of the flawless stainless steel stand. The gauge was then set to zero. An empty shell was then positioned on top of the stainless-steel stand. To measure the shell’s thickness, the hypodermic needle was introduced into the shell aperture and allowed to touch the point inside the shell that was closest to the orange dot, which represents where there was contact between the external side of the outer shell lip and the stainless-steel stand. The green arrow indicates the vertical movement of the needle attached to the dial gauge, which was needed to introduce the needle inside the shell (Fig. S2).

Microgeographic divergence between the two ecotypes as defined by Richardson *et al.* (2014) was then calculated for all traits that differed significantly between the two ecotypes. Standardized spatial scale of divergence and adaptation between the sampled “population” of each ecotype was estimated in Wrights, where a Wright is defined as the number of standard deviations of the focal trait separating two ecotypes within the lifetime dispersal neighborhood length of a snail (Richardson *et al.* 2014). Two published estimates were used for median vertical displacement of 1.5m and the highest median upshore displacement of 2m (Erlandsson *et al.* 1998). These median values were multiplied by 2.8 to convert them to dispersal distance, *d* (Wright 1969). A distance of 20 m between the habitats containing the two pure populations of ecotypes was assumed (Johannesson *et al.* 1993). Divergence, W, is defined as:

$$W = \frac{(\bar{z}_c - \bar{z}_w)d}{h\sqrt{V}} \qquad S1$$

where $\bar{z}_c$ is the trait mean for the crab ecotype, $\bar{z}_w$ for the is the trait mean for the crab ecotype, ,

V is their pooled sample variance, *d* is the dispersal distance and *h* is the distance between pure wave ecotype habitat and pure crab ecotype habitat (Richardson *et al.* 2014). Divergence between two ecotypes within one dispersal neighborhood that was significantly greater than zero standard deviation units per metre was classified as microgeographic divergence (Richardson *et al.* 2014).
