# Supplementary material for: Size selection by a gape‐limited predator of a marine snail: Insights into magic traits for speciation
Source: Ecol Evol. 2016 Dec 20;7(2):674–88. doi: 10.1002/ece3.2659 (PMC5243190; doi:10.1002/ece3.2659)
Supplement: Supplementary file 3 [file ECE3-7-674-s003.pdf]

## APPENDIX 3. EXTENDED METHODS MODEL PARAMETERIZATION

## DETAILS OF MODEL PARAMETERIZATION

Parameters for equation (1) were estimated using similar field and statistical methods to those described in (Boulding *et al.* 2007). The median dispersal distances for wave ecotypes transplanted back to the low shore at Silleiro was estimated at 2 m/month and that for the crab ecotype transplanted back to the high shore at a different site was estimated at 1.5 m/month (Erlandsson *et al.* 1998) and these were converted to d by multiplying by 2.8 (Wright 1969). When transplants were done back to exact location from which they had been collected in northern Spain, the mean displacements were smaller ranging from 0.132 to 0.301 m/week for the crab ecotype and from 0.021 to 0.066 m/week for the wave ecotype (Cruz *et al.* 2004b). We assumed the sample mean was close to the sample median and converted to them to d by multiplying by 2.8. The heritability estimates used for shell weight were those from the literature (range 0.1 to 0.5; average 0.3; see Boulding and Hay 1993; Carballo *et al.* 2001; Conde-Padín *et al.* 2007; Galindo *et al.* 2013). To estimate $\omega_c$, the strength of stabilizing selection required assuming that fitness could be described by a Gaussian Fitness function for which we knew all other parameters.

We used equation 2 to estimate the differences in the optima, θ, and the standard deviation, $\omega_c$, of a Gaussian fitness function representing the fitness of *L. saxatilis* of different sizes or ecotypes in the presence of crabs, we assume that the large crab ecotype has an optimal size, shape and shell thickness for an environment with predatory crabs. We transformed our empirical parameter estimates into phenotypic standard deviations (PSD). This allows our modelling and empirical results to be easily compared with each other and with previous purely theoretical studies (Boulding and Hay 2001). For example, to transform calculations involving

"shell length" parameters in mg to PSD, we divided by the standard deviation for shell length of the 4-mm crab ecotype of *L. saxatilis* (Table 1).

For example, for the second tethering experiment we assumed that snails of the 4-mm crab ecotype had a shell thickness close to the optimum for the high shore habitat where crabs were abundant, $z_x = z_c = \theta$ (Boulding *et al.* 2007); therefore, $\Delta\theta = (z_x - \theta) = 0$ and that snails of the 4-mm wave ecotype $z_x = z_w$ so that their deviation from the optimum $\Delta\theta = (z_w - z_c)$, all in PSD units. $W_x$ and $z_x$ values from all three High transect levels of the second tethering experiment were used to estimate $\omega$ using the nonlinear least squares regression in Systat with the Quasi-Newton method option. Only the binary survival data for the High transect level was used in this estimate because shell-breaking predation was essentially zero at the Mid level for transects T2 and T3 and zero at all three transects at the Low level.
