# Supplementary material for: Size selection by a gape‐limited predator of a marine snail: Insights into magic traits for speciation
Source: Ecol Evol. 2016 Dec 20;7(2):674–88. doi: 10.1002/ece3.2659 (PMC5243190; doi:10.1002/ece3.2659)
Supplement: Supplementary file 4 [file ECE3-7-674-s004.pdf]

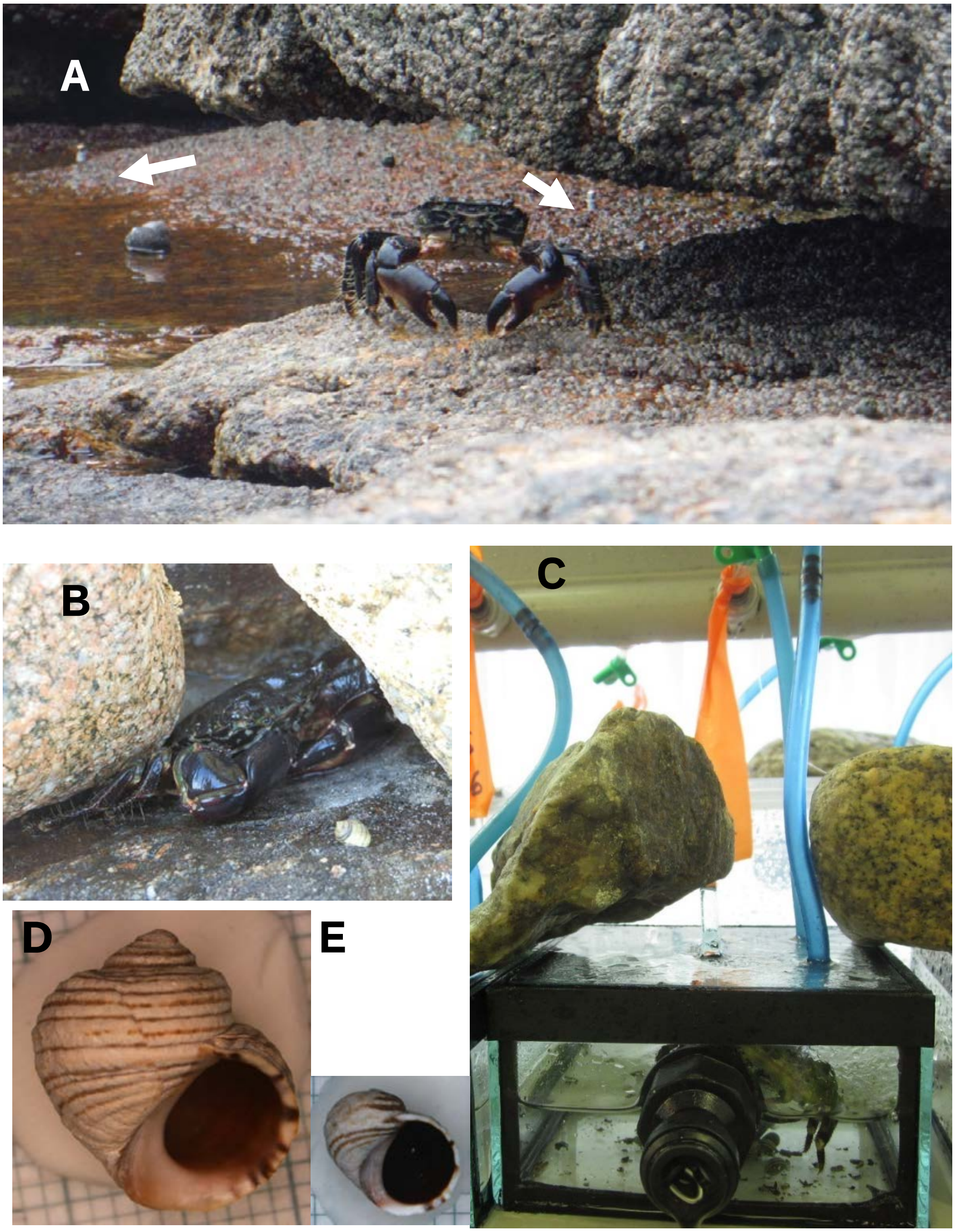

Figure S1. The marbled shore crab *Pachygrapsus marmoratus* photographed:
(A) near transect T2 High (Fig. 1) white arrows indicate tethering screws,
(B) near University of Vigo's Estación de Ciencias Mariñas de Toralla (ECIMAT),
(C) in individual aquaria - each with an independent flow-through water supply that were used for laboratory predation experiments at ECIMAT.
(D) Crab ecotype of *Littorina saxatilis* oriented for geometric morphometric analysis,
(E) Wave ecotype of *Littorina saxatilis* oriented for geometric morphometric analysis.

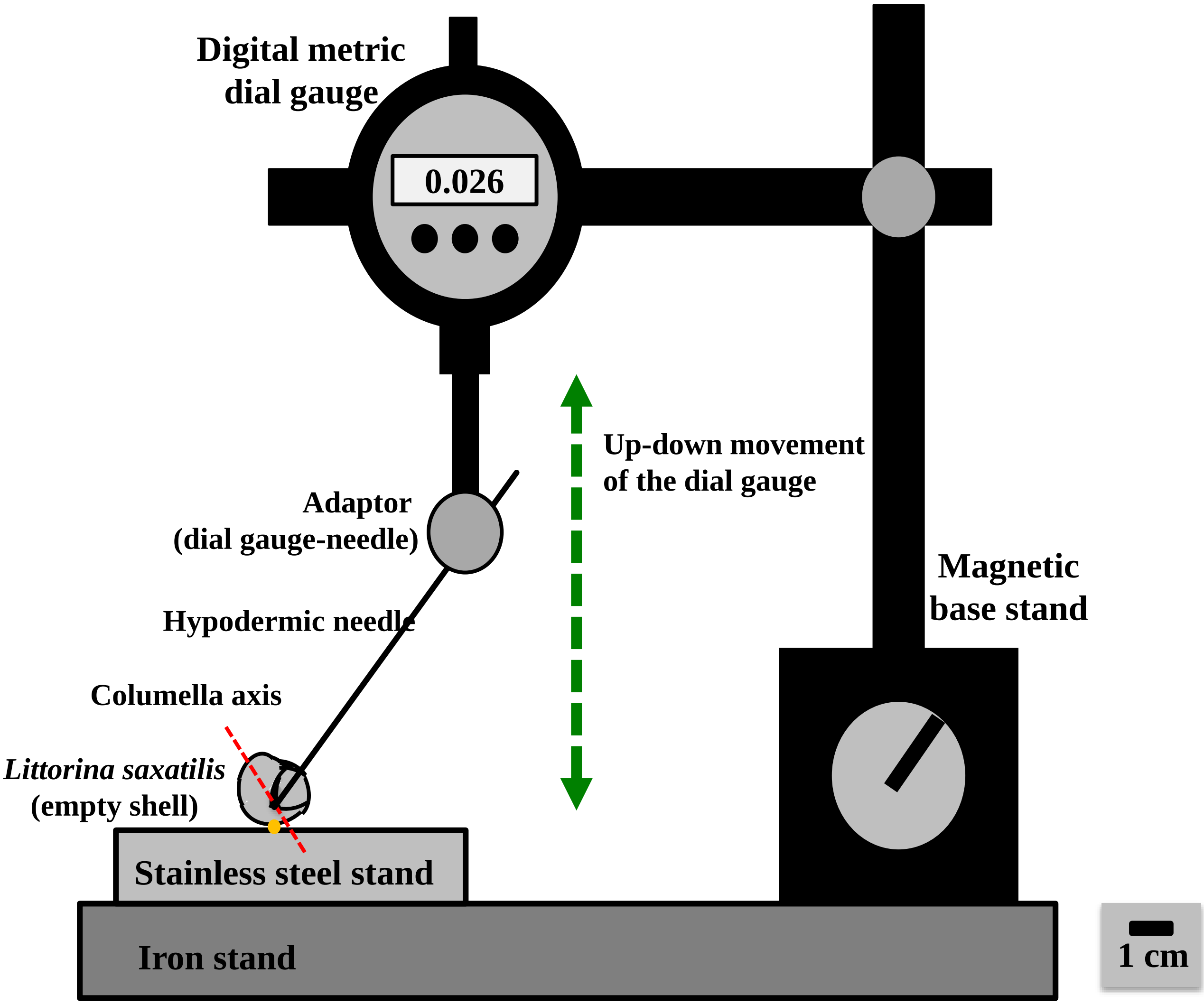

Figure S2. Scheme representing the metric dial gauge used to measure the thickness of the empty shells of the littorinid snail, *L. saxatilis* (Appendix 2).

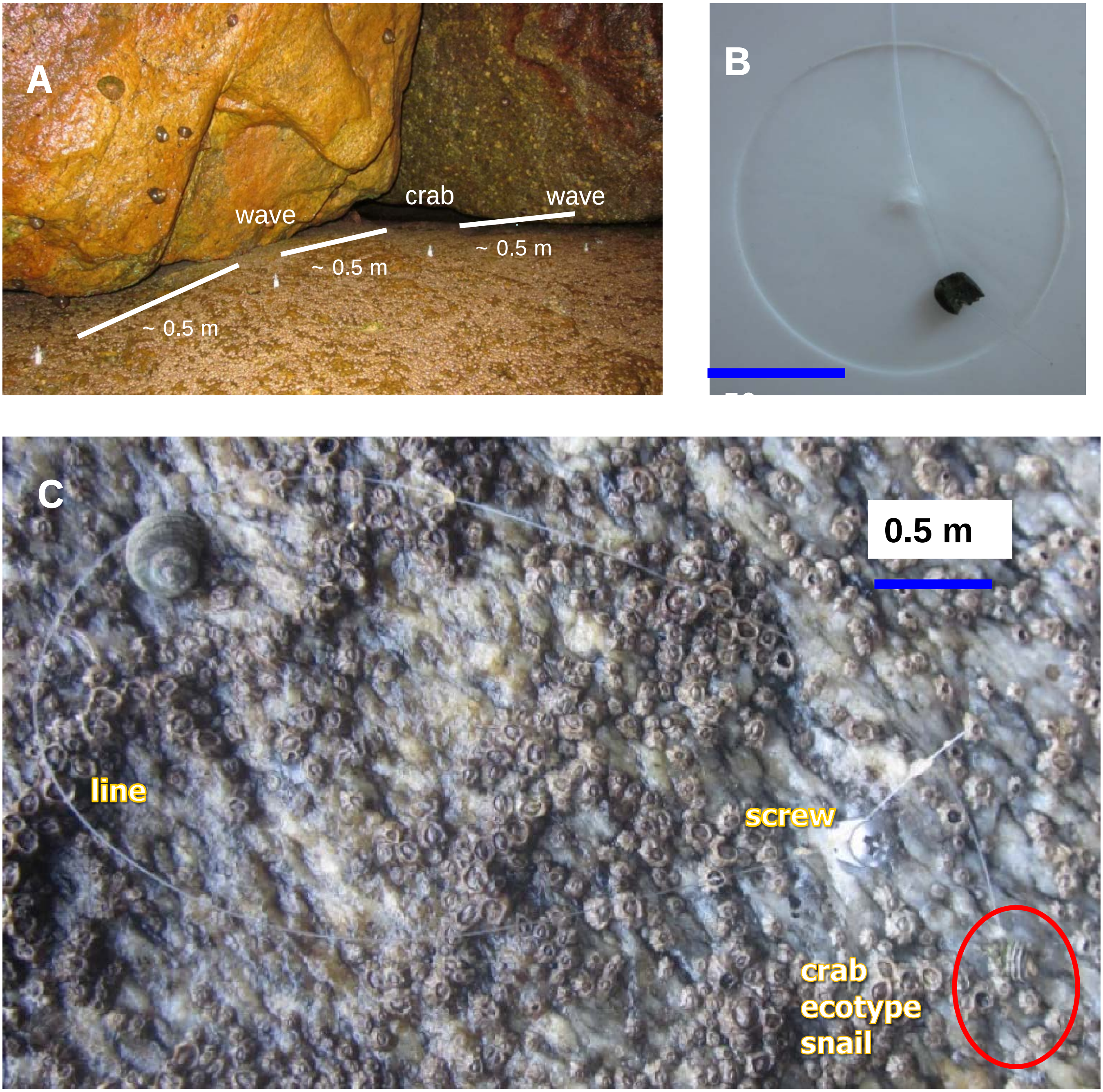

Figure S3. Tethering Methods (A) Screws on the rocky shore separated by 0.5 m. The two ecotypes were tethered alternately to successive screws at each level of the 3 transects. (B) Shell fragment still attached with epoxy to the knot on the fishing line that tethered it to a screw classified as “shell fragment” (SF). (C) Crab ecotype 4mm snail attached to the fishing line that tethers it to a screw classified as “alive undamaged”(AU).

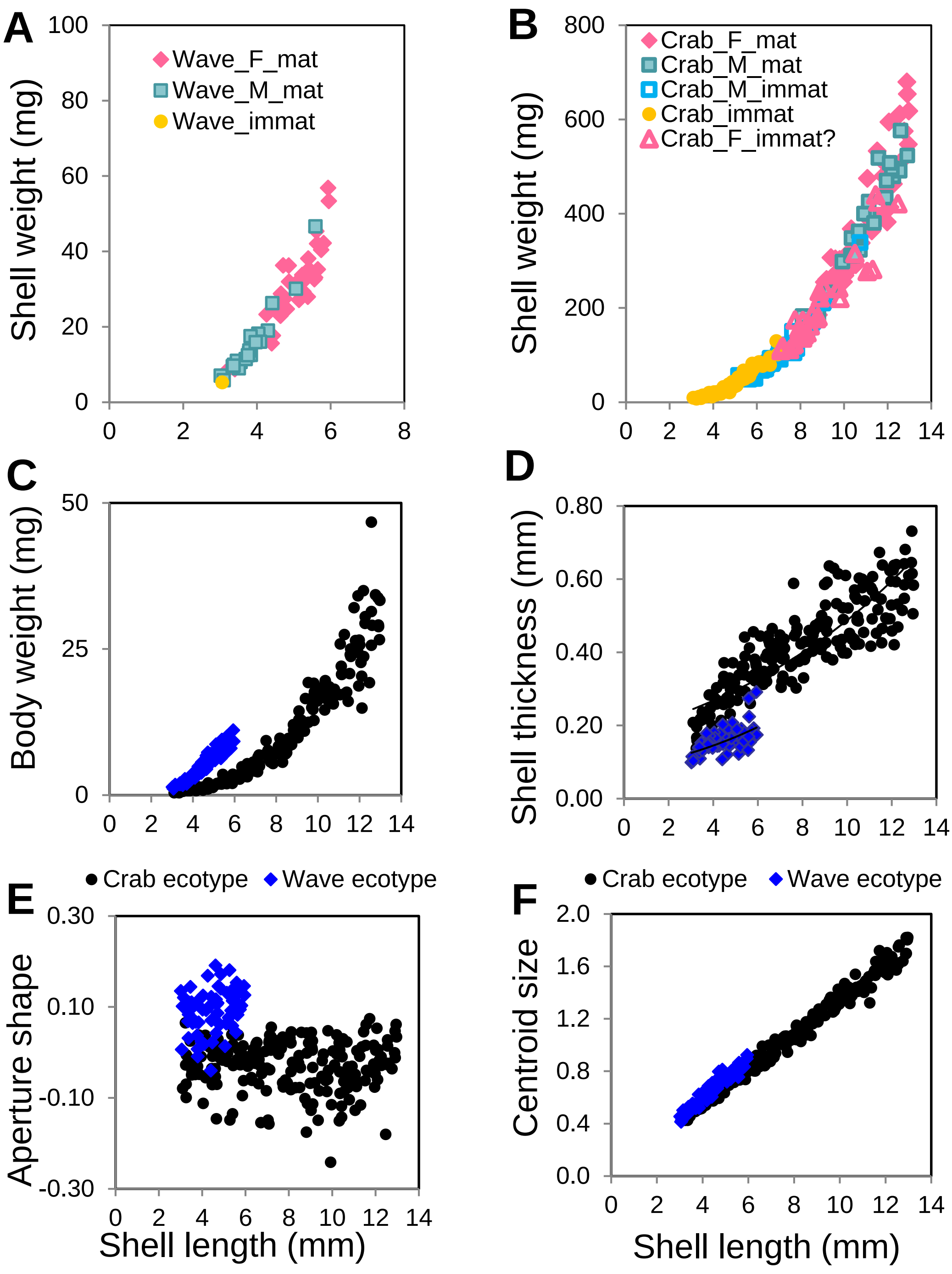

Figure S4. Phenotypic traits of the two ecotypes as a function of shell length.

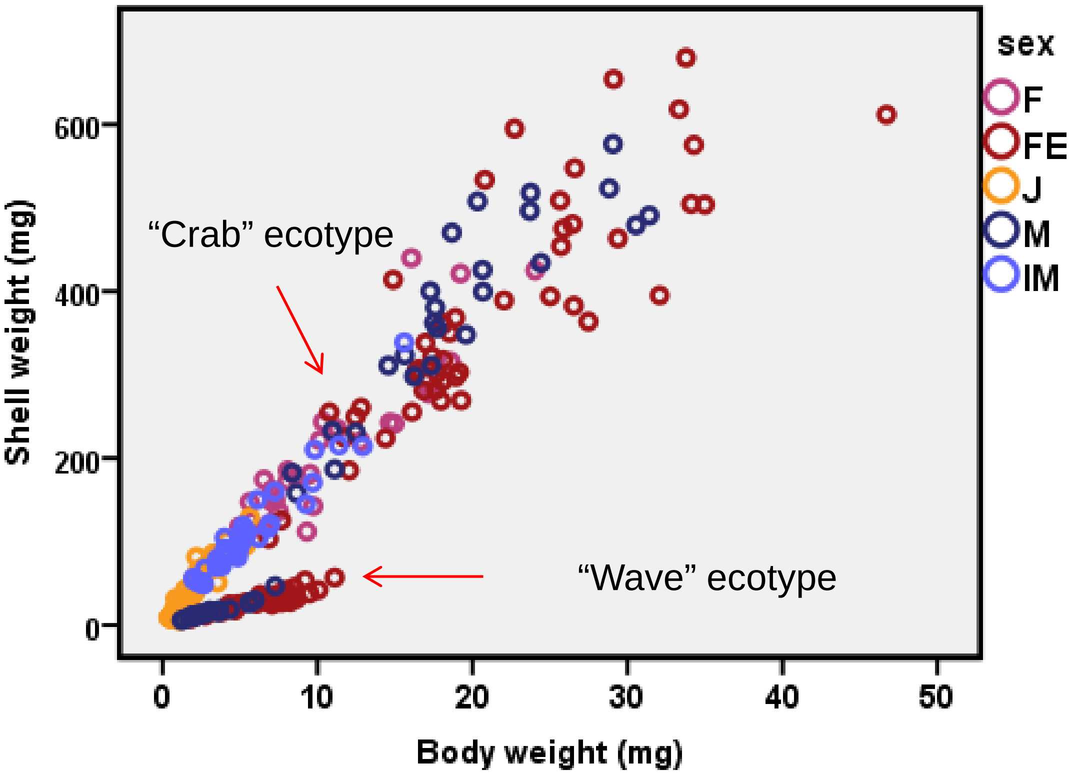

Figure S5. Shell weight (y) as a function of dried body weight (x) with sexually mature individuals indicated for each ecotype (F=female?, FE=female with embryos, J=juvenile, M=mature males with penes, IM=immature males with bump).

Linear regression equations (mg):

crab ecotype: $y = 17.18x + 17.02$, $R^2 = 0.926$;

wave ecotype: $y = 4.33\ x + 1.773$, $R^2 = 0.886$.

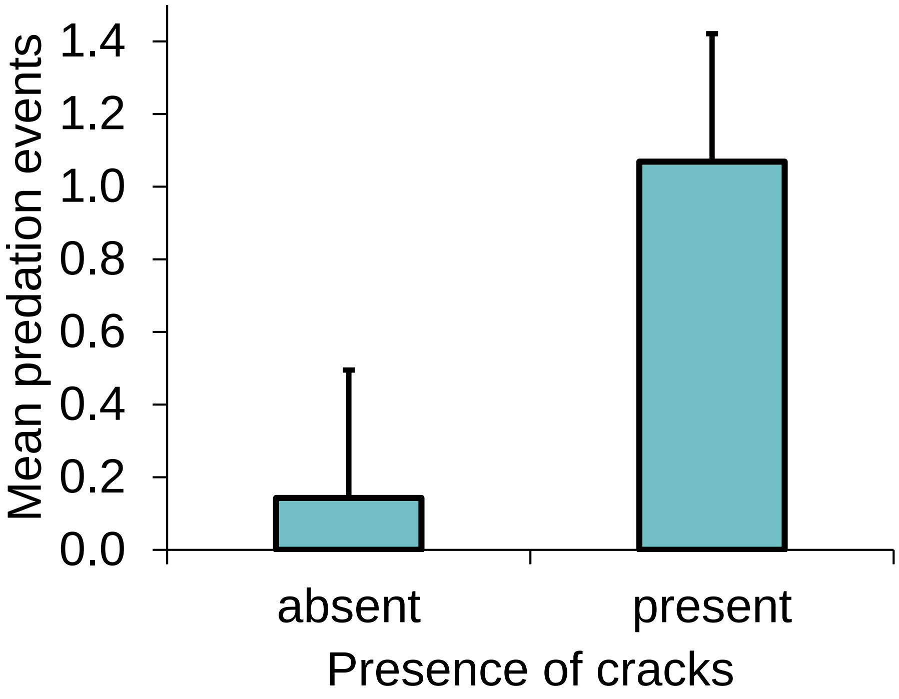

Figure S6. Effect of proximity to a crack on mean number of predation events in first tethering experiment (using the “crab” ecotype of Littorina saxatilis of the 4-mm or 9-mm prey categories).

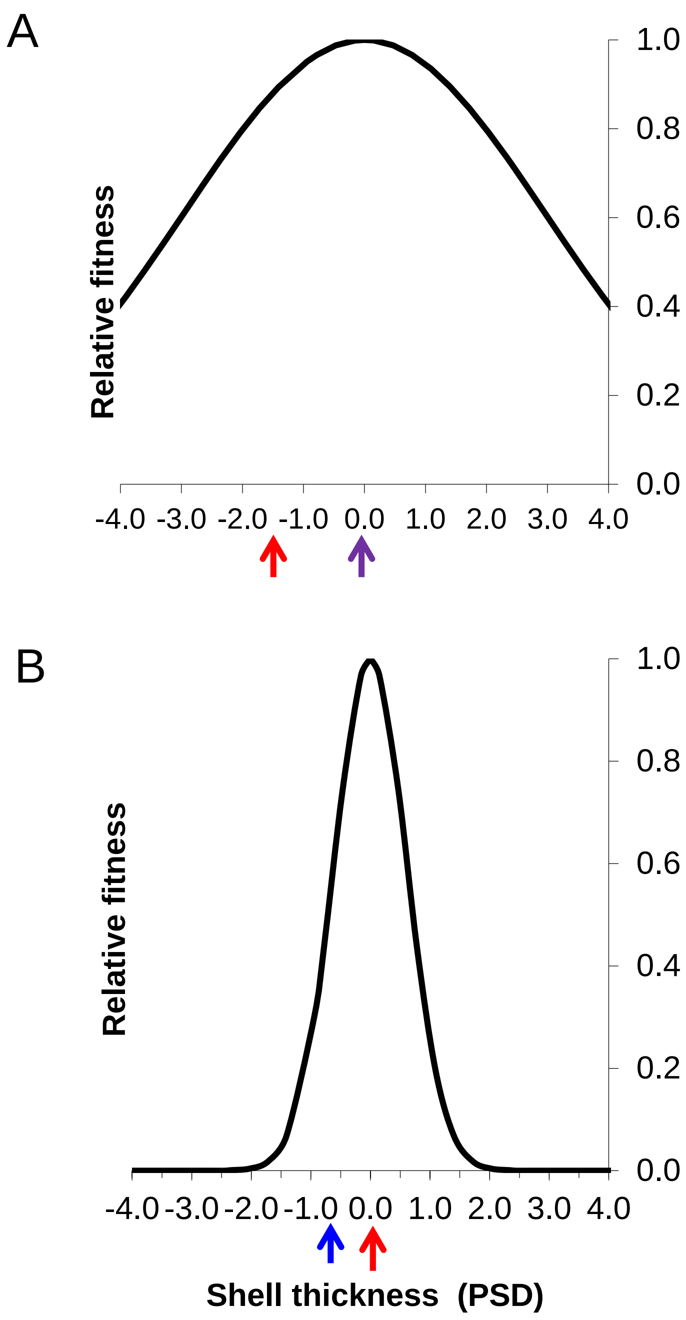

Figure S7. Estimate of standard deviation of fitness function, ω, from shell trait measurements (Table 1) and survival in field tethering experiments (Table 4)**.**
(A) Relative fitness of small crab ecotype (Δθ shell length = –1.38 PSD, red arrow) and large crab ecotype (Δθ shell length = 0.0, purple arrow) in upper intertidal zone during the first tethering experiment. Standard deviation of estimated fitness function, ω = 2.72 PSD**.**
(B) Relative fitness of small wave ecotype (Δθ shell thickness = –0.279, blue arrow) and crab ecotype (Δθ shell thickness= 0, red arrow) in upper intertidal zone during second tethering experiment. Standard deviation of fitness function, ω = 0.549 PSD.
