# Supplementary material for: Size selection by a gape‐limited predator of a marine snail: Insights into magic traits for speciation
Source: Ecol Evol. 2016 Dec 20;7(2):674–88. doi: 10.1002/ece3.2659 (PMC5243190; doi:10.1002/ece3.2659)
Supplement: Supplementary file 5 [file ECE3-7-674-s005.pdf]

SUPPLEMENTARY TABLES:

Table S1: General Linear Model (GLM) to test for difference among ecotypes in the slope of the linear regression lines with dry shell weight (mg) as the dependent variable and dry body weight (mg) as the independent variable.

Source	Type III Sum of Squares	df	Mean Square	F	P value
Model	1233827.[a]	4	308456.924	1540.112	.000
ecotype	617.287	2	308.644	1.541	.217
bodyweight	177372.	1	177372.332	885.612	.000
ecotype x bodyweight	71717.	1	71717.874	358.084	.000
Error	35449.947	177	200.282		
Total	1269277.	181			

[a] Note that intercept was not included in the model so that the differences in the slopes could be compared more easily using the interaction between ecotype and shell weight.

Table S2. Logistic regression analysis of the survival of the crab ecotype (4-mm and 9-mm size classes) in laboratory experiment 1 as the dependent variable using the SPSS procedure GENLIN. The predictor variables were snail size-class with crab claw height (CH) as the covariate. Assumptions were that the probability distribution is Binomial, the link Function is Logit and that the reference category for the response variable fate was alive. Note especially that the presented model with an interaction between claw height and snail_size-class fit the data better (AIC=898.5) than the same model without the interaction term (AIC=1986.5). Adding claw height squared (CH^2) to the model main effects did not improve the fit (AIC=898.8).

Tests of Model Effects

Source	Type III		
	Wald Chi-Square	df	P value
(Intercept)	261.669	1	.000
snail_sizeclass	35.956	1	.000
snail_sizeclass * CH	427.934	2	.000

Dependent Variable: fate
Model: (Intercept), snail_sizeclass, snail_sizeclass * CH

Estimates of proportion of snails preyed upon by crabs

snail_sizeclass	Mean	Std. Error	95% Wald Confidence Interval	
			Lower	Upper
9mm	.00	.001	.00	.01
4mm	.56	.019	.53	.60

Covariates appearing in the model are fixed at the following values:
CH=10.2

Table S3. Selection differential, S, and standardized univariate linear selection gradient, $ß_1$, estimates for shell length for 7 replicates of laboratory experiment 1 with 4-mm and 9-mm size classes of the crab ecotype. See Appendix 1 for detailed methodology.

crab#	Carapace width (mm)	Claw height (mm)	S (mm)	SD	N	ß1±SE	ß1 C.I.
1	29.5	12.1	0.725	0.7897	7	0.23±0.04	0.16-0.30
2	22.5	9.16	1.487	0.6560	7	0.56±0.04	0.47-0.64
3	23.0	8.42	0.955	0.8359	7	0.32±0.04	0.24-0.40
4	25.4	9.60	0.466	0.5992	7	0.15±0.03	0.09-0.21
5	22.4	9.02	0.673	0.7298	7	0.24±0.04	0.16-0.31
6	22.5	8.77	0.333	0.4733	7	0.11±0.03	0.06-0.16
7	23.6	9.83	1.978	0.9362	7	0.69±0.04	0.61-0.77
8	29.2	11.6	2.500	0	7	1.00±0.02	0.96-1.04
9	33.1	14.1	2.500	0	7	1.00±0.06	0.88-1.12
10	35.5	15.5	2.500	0	7	1.00±0.11	0.79-1.21
11	22.7	9.16	0.417	1.021	6	0.08±0.03	0.03-0.14
12	19.0	6.63	0.089	0.2362	7	0.03±0.01	0.00-0.06
13	19.7	7.43	0.240	0.1995	6	0.09±0.03	0.04-0.14
14	17.6	6.81	0.000	0	7	0.00	-
15	19.0	7.93	0.194	0.2349	6	0.07±0.02	0.02-0.12
16	17.3	5.98	0.000	0	7	0.00	-

17	26.9	10.9	2.143	0.9449	7	0.75±0.04	0.68-0.82
18	30.6	9.9	0.364	0.5844	7	0.11±0.03	0.06-0.16
19	36.2	16.5	1.964	0.9835	7	0.91±0.18	0.55-1.27
20	32.0	13.5	2.500	0	7	1.00±0.03	0.95-1.05

Table S4. Logistic regression with the survival of the crab and wave ecotypes (4-mm size class) in laboratory experiment 2 as the dependent variable using the SPSS procedure GENLIN. Predictor variables were ecotype with claw height (CH) and claw height squared (CH^2) as the covariates. Assumptions were that probability distribution is Binomial, the link Function is Logit and that the reference category for the response variable fate was Alive. The presented model with the square of claw height, claw height, and the interaction between claw height and snail ecotype fit better (AIC= 339.8) than the same model without the interaction term (AIC=342.6). Most notably, the omission of the square of claw height from the model reduced the fit and slowed the convergence of the model (AIC=358) even with the interaction added (AIC=359.6).

Tests of Model Effects

Source	Type III		
	Wald Chi-Square	df	Sig.
(Intercept)	.333	1	.564
Ecotype	.246	1	.620
CH^2	17.760	1	.000
CH	2.311	1	.128
Ecotype * CH	2.711	1	.100

Dependent Variable: fate Model: (Intercept), Ecotype, CH^2, CH, Ecotype * CH

Estimates of proportion of snails preyed upon by crab

Ecotype	Mean	Std. Error	95% Wald Confidence Interval	
			Lower	Upper
crab	.32	.033	.26	.39
wave	1.00	.000	.99	1.00

Covariates appearing in the model are fixed at the following values: CH=9.71; CH^2=101.0

Table S5. Selection differential, S, and univariate linear selection gradients, ß$_1$, for N replicates of laboratory experiment 2 with 4-mm size classes of crab and wave ecotypes. See Appendix 1 for detailed methodology.

crab#	Carapace width (mm)	Claw height (mm)	S (mm)	SD	N	ß$_1$±SE	ß$_1$ 95%C.I.
1	29.5	12.1	0.0504	0	4	1.00±0.04	0.93-1.07
2	22.5	9.2	0.0518	0.00280	4	1.03±0.05	0.93-1.12
3	23.0	8.4	0.0511	0.00133	4	1.01±0.04	0.94-1.09
4	25.4	9.6	0.0511	0.00133	4	1.01±0.05	0.90-1.12
5	22.4	9.0	0.0504	0	4	1.00±0.05	0.91-1.09
6	22.5	8.8	0.0511	0.00133	4	1.01±0.05	0.92-1.11
7	23.6	9.8	0.0504	0	4	1.00±0.10	0.81-1.20
8	29.2	11.6	0	0	4	0.00	-
9	33.1	14.1	0	0	4	0.00	-
10	35.5	15.5	0	0	4	0.00	-
11	22.7	9.2	0.0511	0.00133	4	1.01±0.00	-
12	19.0	6.6	0.0504	0	4	1.00±0.05	0.89-1.11
13	19.7	7.4	0.0558	0.00639	4	1.11±0.09	0.92-1.29
14	17.6	6.8	0.0397	0.0124	4	0.92±0.03	0.85-0.98
15	19.0	7.9	0.0533	0.00420	4	1.06±0.05	0.95-1.16
16	17.3	6.0	0.0454	0.00823	4	0.95±0.07	0.82-1.09
17	26.9	10.9	0.0504	0	3	1.00±0.22	0.57-1.43

18	30.6	9.9	0.05040	0	1	1.00±0.11	0.77-1.23
19	36.2	16.5	0	0	1	-	-

Table S6: Tethering experiment 1, which used the crab ecotype (4 mm vs 9 mm) in November 2013. Likelihood ratio chi-square for independence (G statistic) is shown separately for analysis type 1, which considered tethers with epoxy only as well as those with shell fragments as being the result of predation (subscripted e) and for analysis type 2, which considered only the tethers with shell fragments still attached as the result of predation (subscripted f).

Interaction Terms	G_e	df_e	P_e value	G_f	df_f	P_f value
TRANSECT x FATE	22.0	2	<0.001	13.195	2	0.001
TIDE_LEVEL x FATE[#]	4.74	1	0.45[§]	1.824	1	0.260[§]
SIZE x FATE*,[#]	20.3	1	<0.001[§]	18.03	1	<0.001[§]

* included in most parsimonious log-likelihood model fitted with SYSTAT: Raftery's BIC = -174.297, LR chi-square = 756.361 df = 142, *P* value > 0.001

[#] included in next most parsimonious log-likelihood model Raftery's BIC = -165.606, LR chi-square = 751.944 df = 140, *P* value < 0.001.

[§] *P* value from Yates Corrected chi-square because df = 1.

Table S7: Tethering experiment 2, which used the crab and wave ecotypes (4-mm prey category) April–June 2014. Likelihood ratio chi-square (G statistic) is shown separately for analysis type 1, which considered tethers with epoxy only as well as those with shell fragments as the result of predation (subscripted e) and for analysis type 2, which considered only the tethers with shell fragments still attached as the result of predation (subscripted f).

Interaction Terms	G_e	df_e	P_e value	G_f	df_f	P_f value
TRANSECT x FATE*	21.097	2	<0.001	2.423	2	0.298
TIDE_LEVEL x FATE*	190.902	2	<0.001	109.408	2	<0.001
ECOTYPE x FATE*	104.803	1	<0.001[§]	86.986	1	<0.001[§]
TIDE_LEVEL x ECOTYPE x FATE[#]	316	5	<0.001	-	-	-

[§] *P* value from Yates corrected chi-square because df = 1.

* term included in the most parsimonious log-likelihood model for analysis 1 fitted with SYSTAT: A hierarchical model with TIDE_LEVEL_CODE x ECOTYPE x FATE had a better fit. LR chi-square = 45.589, df = 24, *P* value = 0.005, Raftery's BIC = -119.664 than a model with just the ECOTYPE x FATE, TRANSECT x FATE, and TIDE_LEVEL x FATE: LR chi-square = 118.662, df = 30, *P* value = 0.000, Raftery's BIC = -87.903 therefore Δchi-square = 118.662 – 45.589 = 73.07, Δdf=6, $p < 0.001$).

[#] For analysis 2, a log-likelihood model with TIDE_LEVEL x ECOTYPE x FATE fit better than a model without the three-way interaction (i.e., LR chi-square = 45.579, df = 24, *P* value = 0.005, Raftery's BIC = -111.987 versus LR chi-square = 374.90, df = 30, *P* value > 0.001, Raftery's BIC = 177.942 therefore Δchi-square = 374.90 – 45.579 = 329.3, Δdf=6, $p < 0.001$).
